# The Extra-Cytoplasmic Function Sigma Factor SigX Modulates Biofilm and Virulence-Related Properties in *Pseudomonas aeruginosa*

**DOI:** 10.1371/journal.pone.0080407

**Published:** 2013-11-18

**Authors:** Gwendoline Gicquel, Emeline Bouffartigues, Manjeet Bains, Virginie Oxaran, Thibaut Rosay, Olivier Lesouhaitier, Nathalie Connil, Alexis Bazire, Olivier Maillot, Magalie Bénard, Pierre Cornelis, Robert E. W. Hancock, Alain Dufour, Marc G. J. Feuilloley, Nicole Orange, Eric Déziel, Sylvie Chevalier

**Affiliations:** 1 Normandie Université, Université de Rouen, Laboratoire de Microbiologie Signaux et Micro-environnement EA 4312, Evreux, France; 2 Centre for Microbal Diseases and Immunity Research, University of British Columbia, Vancouver, Canada; 3 IUEM, Université de Bretagne-Sud (UEB), Laboratoire de Biotechnologie et Chimie Marines EA 3884, Lorient, France; 4 Cell Imaging Platform of Normandy (PRIMACEN), IRIB, Faculty of Sciences, University of Rouen, Mont-Saint-Aignan, France; 5 Department of Bioengineering Sciences, Research group Microbiology, VIB Department of Structural Biology, Vrije Universiteit Brussel, Brussels, Belgium; 6 INRS-Institut Armand-Frappier, Laval, Québec, Canada; University of Oklahoma Health Sciences Center, United States of America

## Abstract

SigX, one of the 19 extra-cytoplasmic function sigma factors of *P. aeruginosa*, was only known to be involved in transcription of the gene encoding the major outer membrane protein OprF. We conducted a comparative transcriptomic study between the wildtype H103 strain and its *sigX* mutant PAOSX, which revealed a total of 307 differentially expressed genes that differed by more than 2 fold. Most dysregulated genes belonged to six functional classes, including the “chaperones and heat shock proteins”, “antibiotic resistance and susceptibility”, “energy metabolism”, “protein secretion/export apparatus”, and “secreted factors”, and “motility and attachment” classes. In this latter class, the large majority of the affected genes were down-regulated in the *sigX* mutant. In agreement with the array data, the *sigX* mutant was shown to demonstrate substantially reduced motility, attachment to biotic and abiotic surfaces, and biofilm formation. In addition, virulence towards the nematode *Caenorhabditis elegans* was reduced in the *sigX* mutant, suggesting that SigX is involved in virulence-related phenotypes.

## Introduction

A common mode of adaptation of bacteria to environmental changes, including those encountered during interactions with host cells, involves the reprogramming of RNA polymerase specificity by activation of alternate sigma factors. In addition to the principal “housekeeping” sigma factor (RpoD or σ^70^ in *Escherichia coli* and *Pseudomonas aeruginosa*), which is responsible for the majority of RNA synthesis in exponentially growing cells, most bacteria possess multiple alternative sigma factors that are used to co-ordinately regulate the expression of genes involved in diverse functions, including stress responses, iron uptake, virulence, morphological development and chemotaxis [1]. The σ^70^ family of sigma factors includes a distinct subfamily of regulators that are activated by signals from the environment and are involved in extra-cytoplasmic functions (ECF), such as secretion, iron transport or stress responses [1]. These ECF sigma factors play thus a key role in the bacterial response to the environment [2].

*Pseudomonas aeruginosa* is a ubiquitous Gram negative bacterium capable of surviving in a broad range of natural environments, although it is best known as a opportunistic human pathogen associated with antibiotic-resistant hospital-acquired infections and as the leading cause of chronic infections contributing to mortality of cystic fibrosis patients [3]. Analysis of the *P. aeruginosa* genome sequence revealed the presence of 19 open reading frames (ORFs) encoding putative proteins with greater than 47% similarity to members of the ECF family [4]. Among these, fourteen are clustered with anti-sigma factors, and twelve also with TonB-dependent transducers [5]. PvdS is found alone, but is well known to be controlled by cell-surface signalling via the FpvA receptor and the FpvR anti-sigma factor. The remaining four ECF sigma factors are not associated with either an anti-sigma factor or a transducer, namely SigX (PA1776), PA1351, PA2896 and PA3285. While no function has been assigned to date to the last three genes, SigX contributes to the transcription of its own gene and is largely responsible for the transcription of the *oprF* gene, which encodes the major outer membrane protein of *Pseudomonadaceae* bacteria [6,7]. OprF is involved in several crucial functions, including cell structure maintenance, outer membrane permeability, environment and host immune system sensing, and virulence [8-11]. SigX shares 49% similarity with the Gram positive bacterium *Bacillus subtilis* SigX and SigW ECF sigma factors, which are induced by alkaline shock, phage infection, salt, and antibiotics affecting cell-wall biosynthesis [12,13]. In *P. aeruginosa*, a *sigX* mutant displayed several phenotypes that could not be restored by complementing with a plasmid-borne copy of *oprF* [6], indicating that SigX is likely involved in the transcriptional regulation of additional genes. To identify the genes that are directly or indirectly SigX-dependent, we performed a transcriptomic study of a *sigX* mutant. These data allowed us to propose and demonstrate several functions that were modulated by SigX in *P. aeruginosa*, including some related to virulence, motility and biofilm formation. 

## Materials and Methods

### Bacterial strains and growth conditions

The bacteria utilized were *P. aeruginosa* H103 (the prototrophic sequenced isolate strain PAO1) [14], its isogenic *sigX* deletion mutant PAOSX, and PAOSX+ in which PAOSX was complemented with the cloned *sigX* gene [7]. Bacteria were grown overnight at 37°C on a rotary shaker (180 rpm) in M9 minimal medium containing 0.2% glucose (M9G). Cultures were inoculated at an initial OD_580_ of 0.08 into fresh M9G medium, and allowed to grow to mid log phase (OD_580_=0.4) before RNA extraction.

### Samples preparation for microarray hybridization

Samples for microarray hybridization were prepared as previously described by Tremblay and Déziel [15], with minor modifications. Briefly, total RNA was prepared by the hot acid-phenol method as previously described [7]. RNA purity was assessed by spectrophotometry (NanoDrop ND-1000). Samples showing A_260_/A_280_ and A_260_/A_230_ ratios above 2.0 were selected. RNA quality was then assessed with a Bioanalyzer 2100 (Agilent Technologies). Samples having a RIN of 9 were retained. Ten μg of total RNA was used for each replicate with random hexamer primers (Invitrogen) and Superscript II reverse transcriptase (Invitrogen) for cDNA synthesis, fragmentation and labeling.

### Microarray hybridization and data analysis

Hybridizations were performed at the Genome Québec Innovation Centre (McGill University, Montréal, Canada). Raw data were corrected for background signals using the RMA algorithm and quantile normalization [16]. 

Raw data were deposited to the Gene Expression Omnibus (GEO) public database (NCBI) under series entry “GSE51076”. Expression levels obtained from three replicates for each condition were compared using the FlexArray 1.3 software [17]. Only genes showing a *p*-value < 0.05 using the Empirical Bayes (Wright and Simon) algorithm were considered further. Since the RMA algorithm decreases the false positive rate and compresses the fold change, a 2-fold change cut-off value was used for the determination of differentially expressed genes. Functional classification and over-representation analyses were performed using the PseudoCAP functional classes (http://www.pseudomonas.com, [18]). Expression data for all differentially expressed genes is available in Table S1.

### Quantitative RT-PCR

Synthesis of cDNAs and real time PCR were performed as previously described [7,11], using primers described in Table S2. PCR reactions were performed in triplicate and the standard deviations were lower than 0.15 Ct. 

### Secreted factor assays

Secreted exotoxin A production was evaluated as previously described by Gaines et al. [19]. Pyocyanin was quantified from supernatants of cultures in King A medium [20]. Briefly, the phenazine pigment was extracted from cell-free culture supernatants with 3 ml of chloroform by vortexing. The chloroform phase was then extracted with 0.2 N HCl, and the absorbance of the aqueous phase was measured at 520 nm. Concentrations, expressed as µg of pyocyanin produced per ml of culture supernatant, were determined as 17.072 X OD_520_ [21]. To measure total siderophore production, bacteria were grown overnight in King *B medium* [20]. Culture supernatants were filtered (0.22 µm) and tested for the absence of bacteria by inoculating 3 mL of LB with an aliquot of supernatant. A fraction of each culture supernatant corresponding to the same bacterial density was applied to wells dug in chrome azurol S (CAS) agar plates [22]. The orange halo diameters were measured after 24h incubation of the plates at room temperature.

### Twitching and swarming motilities

Twitching and swarming motilities were assayed on nutrient agar plates containing 1% or 0.5% agar (w/v) (AES Chemunex, Bruz, France), respectively. For twitching assays, overnight cultures in M9G medium were diluted to an OD_580_ of 0.4 and 5 µL were used to inoculate M9G plates underneath the agar layer. After 24h of incubation at 37°C, the agar was removed and the cells adhering to the Petri dish were stained with 0.4 % crystal violet. For swarming assays, 5 µL of bacterial suspension from an overnight culture grown in M9G containing 1% casamino acids (M9GCAA, Difco, France) as a nitrogen source, were spotted on the surface of a 0.5% agar containing M9GCAA plate, and incubated for 24h at 37°C.

### Cellular culture and cell adherence assays

The Caco-2/TC7 clonal cell line, derived from parental Caco-2 cells [23], was used in this study. TC7 cells differ from the parental Caco-2 cells in their shorter population doubling time and higher cell density leading to fully differenciated cells after a shorter period of time [23]. The Caco-2/TC7 clonal cell line was kindly provided by A. Servin (INSERM, UMR-S756, Châtenay-Malabry, France) at passage 23 and was used between passages 25 and 35. Cells were routinely grown at 37°C in a controlled atmosphere containing 5% CO_2_ in Dulbecco’s modified essential medium (DMEM) supplemented with 15% heat inactivated fetal calf serum (FCS) and 1% nonessential amino acids. The medium was changed three times a week. The adhesive behavior (binding index) of *P. aeruginosa* strains onto intestinal Caco-2/TC7 cells was investigated using the procedure described by Picot et al. [24], adapted to *P. aeruginosa*. Cells were allowed to adhere onto eukaryotic cells for 3h.

### Static biofilm assays

Overnight cultures were diluted in LB to an OD_580_ of 0.08. Three ml of this bacterial suspension was added into borosilicate glass tubes and incubated at 37°C for 24 h without shaking. Pellicles were observed at the air-liquid interface of the culture. To assay the solid surface associated biofilm formation, the standing culture was then removed and the tubes were washed gently twice with distilled water. Then 4 ml of 0.4% crystal violet (v/v) was added into each tube and allowed to stand at room temperature for 20 min before residual stain was removed. After washing with distilled water, the stained biofilms were dissolved in 3 mL of 100 % ethanol and absorbance measured at 595 nm. 

### Adherence assay

For adherence onto glass slides, mid-log phase bacteria transformed with pSMC21 plasmid containing the gene encoding the green fluorescent protein [25] were diluted in 0.9% NaCl to an OD_580_ of 0.6 and allowed to adhere for 2h at 37°C. Attached cells were observed using a confocal laser scanning microscope (Zeiss, Brucker, Germany). 

### *Caenorhabditis elegans* infection model

Experimental procedures and data analysis were performed as previously described [11]. Briefly, *C. elegans* wild-type Bristol strain N2 worms were grown at 22°C on nematode growth medium (NGM) agar plates using *E. coli* OP50 as the nutrient. Bacteria were grown as described above in M9G broth overnight and harvested, and 10^9^ bacteria were spread onto NGM solidified agar plates and incubated at 37°C overnight. The lawn that covered the NGM agar plates was homogeneous and visually similar for each strain tested. The plates were cooled to room temperature for 4 h, and 20 to 30 L4-synchronized worms were placed on the plates and incubated at 22°C. Worm survival was scored daily for 12 days using an Axiovert S100 optical microscope (Zeiss, Oberkochen, Germany) equipped with a digital camera (DXM 1200F; Nikon Instruments, Melville, NY). An assay consisted of three independent replicates (plates). The virulence value of each bacterial strain was the mean of three independent assays. For killing assay, nematode survival was calculated by the Kaplan-Meier method, and the significance of survival differences was tested using the log rank test (Prism software, version 4.0; GraphPad Software, San Diego, CA). 

## Results

### Microarray analysis

A microarray analysis was conducted comparing the wildtype *P. aeruginosa* strain H103 to its isogenic mutant PAOSX [7] in M9G medium, both showing similar growth under these conditions (Figure S1). Since the contribution of SigX to *oprF* transcription is maximal during exponential growth phase [7], and since a higher activity during this phase is a common feature of many ECF sigma factors [26], we performed this transcriptomic study on cells grown to mid exponential phase (OD_580_ = 0.4 ± 0.1). Analysis of three independent experiments comparing the H103 and PAOSX strains revealed a total of 307 differentially expressed genes (*p*<0.05 by Empirical Bayes statistical test) that differed by more than 2 fold (Table S1). Of these genes, 153 were down-regulated (i.e. positively regulated by SigX under the given condition), while 154 were up-regulated. 

Twenty four genes that were differentially expressed between the wildtype H103 and the *sigX* mutant strain were selected for validation using qRT-PCR (Table 1), including *sigX* and *oprF*, a previously confirmed SigX gene target [6,7]. The comparison of expression data from microarrays and qRT-PCR demonstrated a very good correlation between the two datasets with a Pearson correlation coefficient of 0.98 (data not shown). These data further confirmed that SigX regulates the expression of its own gene, a common feature of stress-responsive ECF sigma factors [26], as well as that of two major outer membrane protein encoding genes, *oprF*, as previously determined, and *oprD*. 

**Table 1:** Selected genes used for validation of the microarray data by qRT-PCR.

**Gene number**	**Gene name**	**Product name and/or function**	**PAOSX/H103 Fold change (log_2_)**	
			**DNA array**	**qRT-PCR**
PA0044	*exoT*	Exoenzyme T	3.9	4.8
PA0085	*hcp1*	Hcp1	2.5	2.8
PA0409	*pilH*	Twitching motility protein PilH	2.5	3.9
PA0527	*dnr*	Transcriptional regulator Dnr	3.9	5.8
PA0779	*asrA*	Aminoglycoside-induced ATP-dependent protease	2.4	2.2
PA0958	*oprD*	Basic amino acid porin OprD	-2.9	-3.0
PA1544	*anr*	Transcriptional regulator Anr	1.6	1.7
PA1546	*hemN*	Oxygen-independent coproporhyrinogen III oxidase	3.1	4.4
PA1148	*toxA*	Exotoxin A precursor	-5.1	-3.9
PA1317	*cyoA*	Cytochrome o ubiquinoloxidase subunit II	-2.8	-2.4
PA1706	*pcrV*	Type III secretion protein PcrV	3.3	5.2
PA1774	*cfrX*	CfrX protein	-2.5	-4.3
PA1775	*cmpX*	Conserved cytoplasmic membrane protein CmpX	-2.9	-1.4
PA1776	*sigX*	ECF sigma factor SigX	-4.2	-2.1
PA1777*	*oprF*	Major porin OprF	-1.5	-2.2
PA2018	*mexX*	Antibiotic resistance and susceptibility	6.7	23.2
PA3006	*psrA*	Transcriptional regulator PsrA	-2.3	-1.6
PA3405	*hasE*	Metalloprotease secretion protein	-3.0	-6.3
PA3479	*rhlA*	Rhamnosyltransferase chain A	-3.4	-3.6
PA3879	*narL*	Two-component response regulator NarL	4.7	5.4
PA4231	*pchA*	Salicylate biosynthesis isochorismate synthase	-2.9	-2.0
PA4296	*pprB*	Two-component response regulator PprB	-2.2	-2.2
PA4306	*flp*	Type IVb pilin Flp	-17.0	-25.6
PA4525	*pilA*	Type 4 fimbrial precursor PilA	-49.1	-76.3

* The SigX target *oprF* has been added to this Table.

**Figure 1:**
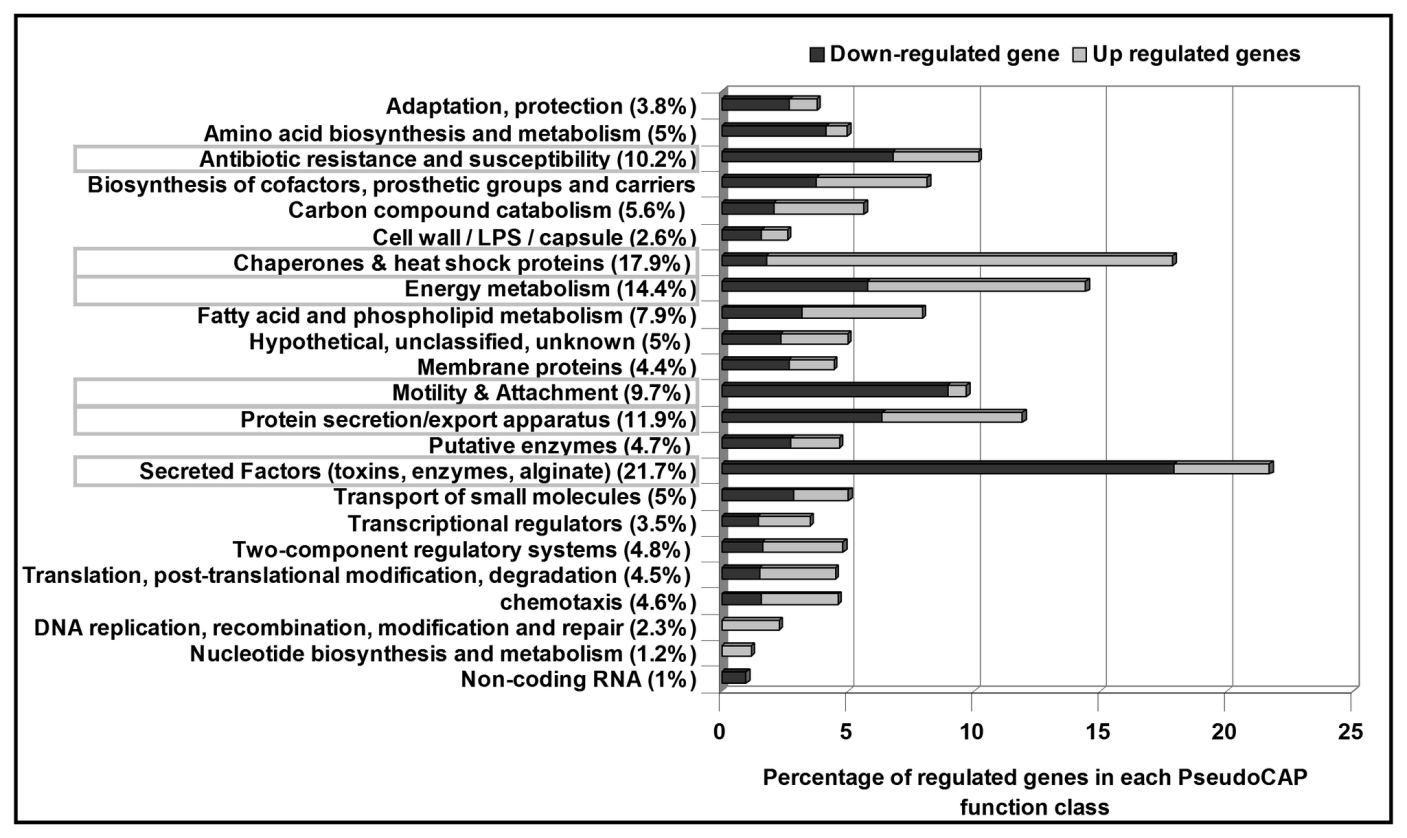
Functional classes of SigX-regulated genes identified by expression profiling on DNA array. All 307 genes that had a significant difference in expression between wildtype and mutant strains (Fold change ≥2,*p*-value ≤0.05 as determined by Empirical Bayes) were included and classified according to their function. Functional classes were determined using the *Pseudomonas* Genome Project website (www.pseudomonas.com; Winsor et al., 2011), among which these framed in grey were discussed.

Based on PseudoCAP analysis [18], most dysregulated genes belong to six functional classes, which represented more than 10% of the total genes in each of these classes (Figure 1). These included the “secreted factors”, and “motility and attachment” classes, in which the large majority of the affected genes were down-regulated in the *sigX* mutant. In contrast, up-regulated genes were mostly found in the “chaperones and heat shock proteins” class. Such trends were not as clear in the “energy metabolism”, “protein secretion/export apparatus”, and “antibiotic resistance and susceptibility” classes in which up- and down-regulated genes were both well represented. We chose to focus subsequent analyses on selected genes from these principal categories (Table 2).

**Table 2:** Selected genes up- and down-regulated in *P. aeruginosa* PAOSX (*sigX* mutant) versus H103 (WT).

**Gene number**	**Gene name**	**Product name and/or function**	**Regulator**	**PAOSX/H103 fold change (log_2_)**
**Motility and Attachment**				
**Flp pilus**				
PA4294	*tadF*	Flp pilus assembly protein TadF		-3.3
PA4299	*tadD*	Flp pilus assembly protein TadD		-4.2
PA4300	*tadC*	Flp pilus assembly protein TadC		-2.8
PA4301	*tadB*	Flp pilus assembly protein TadB		-2.3
PA4302	*tadA*	TadA ATPase		-2.7
PA4303	*tadZ*	Flp pilus assembly protein, TadZ		-2.0
PA4304	*rcpA*	RcpA		-2.3
PA4305	*rcpC*	RcpC		-2.2
PA4306	*flp*	Type IVb pilin Flp	PprB	-17.0
**Cupin**				
PA4648	*cupE1*	Pilin subunit CupE1	PprB	-4.4
PA4651	*cupE4*	Pili assembly chaperone CupE4		-3.2
PA4653	*cupE6*	Adhesin-like protein CupE6		-1.9
**Type IV pilus**				
PA4525	*pilA*	Type 4 fimbrial precursor PilA		-49.1
PA0408	*pilG*	Twitching motility protein PilG		2.8
PA0409	*pilH*	Twitching motility protein PilH		2.5
PA0410	*pilI*	Twitching motility protein PilI		2.3
**Antimicrobial resistance and susceptibility genes and associated genes**				
PA0779	*asrA*	AsrA		2.4
PA2018	*mexY*	Resistance-Nodulation-Cell Division multidrug efflux transporter		6.7
PA2019	*mexX*	Resistance-Nodulation-Cell Division multidrug efflux transporter		13.3
PA5470	*prfH*	Probable peptide chain release factor PrfH		7.8
PA5471	*---*	Hypothetical protein		6.2
**Stress response related proteins**				
PA1596	*htpG*	Heat shock protein HptG		3.4
PA1597	---	Hypothetical protein		2.1
PA1789	*uspL*	Universal stress related protein UspL	Anr	3.2
PA3911	---	Hypothetical protein		2.2
PA4328	*uspM*	Universal stress related protein UspM	Anr	4.0
PA4352	---	Hypothetical protein	Anr	4.1
PA4385	*groEL*	GroEL protein		2.1
PA4386	*groES*	GroES protein		2.1
PA4542	*clpB*	ClpB protein		2.9
PA4761	*dnaK*	DnaK protein		2.3
PA4762	*grpE*	GrpE protein		4.2
PA5027	*uspO*	UspO protein	Anr	2.3
PA5053	*hslV*	Heat shock protein HslV		4.4
PA5054	*hslU*	Heat shock protein HslU		3.2
PA5440	---	Probable peptidase		2.3
**Iron uptake**				
PA0781	*---*	TonB-dep hemoglobin receptor family protein		-7.7
PA1301	*---*	Probable transmembrane sensor	Fur	-2.7
PA2033	*viuB*	Siderophore interacting protein ViuB	Fur	-5.5
PA2034	*---*	Hypothetical protein	Fur	-4.1
PA4156	*fvbA*	TonB receptor FvbA		-5.5
PA4218	*ampP*	AmpP	Fur/PchR	-3.1
PA4219	*ampO*	AmpO	Fur/PchR	-3.0
PA4220	*---*	Hypothetical protein	Fur/PchR	-4.1
PA4221	*fptA*	Fe(III) pyochelin outer membrane receptor protein	Fur/PchR	-4.0
PA4225	*pchF*	Pyochelin synthase	Fur/PchR	-2.5
PA4226	*pchE*	Dihydroaeruginoic acid synthetase	Fur/PchR	-3
PA4228	*pchD*	Pyochelin biosynthesis protein PchD	Fur/PchR	-3.4
PA4229	*pchC*	Pyochelin biosynthetic protein PchC	Fur/PchR	-3.3
PA4230	*pchB*	Salicylate biosynthesis protein PchB	Fur/PchR	-3.0
PA4231	*pchA*	Salicylate biosynthesis isochorismate synthase	Fur/PchR	-2.9
PA4570	*---*	Hypothetical protein	Fur	-5.9
**Secretion systems and related secreted proteins**				
**Type 1 secretion systems**				
PA1245	*aprX*	AprX		-2.9
PA1246	*aprD*	Alkaline protease secretion protein AprD		-2.0
PA1249	*aprA*	Alkaline metalloproteinase precursor		-3.1
PA1874	*bapA*	Biofilm associated adhesin	PprB	-4.5
PA1875	*bapB*	Outer membrane protein		-2.5
PA1876	*bapC*	ATPase component, ABC transporter		-2.4
PA1877	*bapD*	Membrane fusion protein, ABC transporter		-2.2
PA3404	*hasF*	Probable outer membrane protein precursor		-1.7
PA3405	*hasE*	Metalloprotease secretion protein		-3.0
PA3406	*hasD*	Transport protein HasD		-3.2
PA3407	*hasAp*	Heme acquisition protein HasAp	Fur	-11.2
PA3408	*hasR*	Heme uptake outer membrane receptor HasR precursor	Fur	-4.2
**Type 2 secretion system (Xcp)**				
PA0572	*pmpA*	Putative metalloprotease		-4.1
PA0852	*cbpD*	Chitin-binding protein CbpD precursor		-2.9
PA1148	*toxA*	Exotoxin A precursor	Fur/PvdS	-5.1
PA2939	*paAP*	Probable minopeptidase		-5.7
PA4175	*piv*	Protease IV	Fur/PvdS	-5.5
**Type 3 secretion system**				
PA0044	*exoT*	Exoenzyme T		3.9
PA1701	*pcr3*	Pcr3		2.6
PA1706	*pcrV*	Type III secretion protein PcrV		3.3
PA1707	*pcrH*	Regulatory protein PcrH		3.2
PA2191	*exoY*	Adenylate cyclase ExoY		2.8
PA3842	*spcS*	Specific *Pseudomonas* chaperone for ExoS, SpcS		3.2
PA3843	*---*	Hypothetical protein		2.8
**Type 6 secretion systems**				
**T6SS-HsiI**				
PA0080	*tssJ1*	TSSJ1		2
PA0083	*tssB1*	TssB1		2.9
PA0084	*tssC1*	TssC1		2.3
PA0085	*hcp1*	Hcp1		2.5
PA0086	*tagJ1*	TagJ1		2.2
PA0090	*clpV1*	ClpV1		2.3
**T6SS-HsiIII**				
PA2360	---	Hypothetical protein		-2.8
PA2366	---	Uricase PuuD		-2.6
PA2367	---	Hypothetical protein		-3.3
PA2368	---	Hypothetical protein		-3.5
PA2369	---	Hypothetical protein		-2.3
PA2370	---	Hypothetical protein		-4.2
PA2371	---	Hypothetical protein		-3.8
PA2373	---	Hypothetical protein		-2.9
**Secreted factors (others)**				
PA1902	*phzD2*	Phenazine biosynthesis proteinPhzD	RpoS	-5.4
PA1903	*phzE2*	Phenazine biosynthesis protein PhzE	RpoS	-5.5
PA1904	*phzF2*	Phenazine biosynthesis protein PhzF	RpoS	-7.2
PA1905	*phzG2*	Probable pyridoxamine 5'-phosphate oxidase	RpoS	-7.8
PA4217	*phzS*	Flavin-containing monooxygenase	RpoS	-3.7
PA3478	*rhlB*	Rhamnosyltransferase chain B		-4.5
PA3479	*rhlA*	Rhamnosyltransferase chain A		-3.3
PA2193	*hcnA*	Hydrogen cyanide synthase HcnA		5.6
PA2194	*hcnB*	Hydrogen cyanide synthase HcnB		2.9
PA2195	*hcnC*	Hydrogen cyanide synthase HcnC		2.5
PA3912	*---*	Hypothetical protein		2.3
PA3913	*---*	Probable protease		4
**Energy metabolism**				
**Aerobic respiration**				
PA0105	*coxB*	Cytochrome c oxidase, subunit II	RpoS	-4.1
PA0106	*coxA*	Cytochrome c oxidase, subunit I	RpoS	-4
PA0107	*---*	Hypothetical protein	RpoS	-3.0
PA0108	*coIII*	Cytochrome c oxidase, subunit III	RpoS	-3.1
PA1317	*cyoA*	Cytochrome o ubiquinol oxidase subunit II	Fur	-2.8
PA1318	*cyoB*	Cytochrome o ubiquinol oxidase subunit I	Fur	-2.8
PA1319	*cyoC*	Cytochrome o ubiquinol oxidase subunit III	Fur	-3.0
PA1320	*cyoD*	Cytochrome o ubiquinol oxidase subunit IV	Fur	-3.0
PA1550	*---*	Hypothetical protein		2.6
PA1551	*---*	Probable ferredoxin		2.9
PA1555	*ccoP2*	Cytochrome c oxidase, cbb3-type, CcoP subunit	Anr	6.1
PA1556	*ccoO2*	Cytochrome c oxidase, cbb3-type, CcoO subunit	Anr	6.7
PA1557	*ccoN2*	Cytochrome c oxidase, cbb3-type, CcoN subunit	Anr	4.7
PA4429	*---*	Probable cytochrome C1 precursor		2.3
PA4431	*---*	Probable iron-sulfur protein		2.2
PA4571	*---*	Probable cytochrome c		4.1
PA4587	*ccpR*	Cytochrome c551 peroxydase precursor		8.1
PA5300	*cycB*	Cytochrome c5		2.2
**Denitrification**				
PA0516	*nirF*	Heme d1 biosynthesis protein NirF		3.1
PA0517	*nirC*	Probable c-type cytochrome precursor		8.0
PA0518	*nirM*	Cytochrome c-551 precursor		8.6
PA0519	*nirS*	Nitrite reductase precursor		13.1
PA0526	*---*	Hypothetical protein		5.0
PA1197	---	Hypothetical protein		4.1
**Transcriptional regulators**				
PA0515	*---*	Probable transcriptional regulator		7,1
PA0527	*dnr*	Transcriptional regulator Dnr		3.8
PA1196	---	Probable transcriptional regulator, nitrogen utilization		6
PA1776	*sigX*	ECF sigma factor		-4.2
PA2020	*mexZ*	Transcriptional regulator MexZ		5.3
PA3006	*psrA*	Transcriptional regulator PsrA		-2.3
PA3879	*narL*	Two-component response regulator NarL	Anr	4.7
PA3899	*---*	Probable sigma-70 factor, ECF subfamily	Fur	-2.4
PA4296	*pprB*	Two-component response regulator PprB	Fur	-2.2

Only significantly (*p*-value<0.05) dysregulated genes are included, and the log_2_ fold change cut-off between PAOSX and H103 strains is 2, except for some genes which are included for discussion. Fur-, Anr- PprB- and RpoS-regulated genes are indicated in Regulator column.

### Role of SigX in motility, attachment to biotic and abiotic surfaces, and biofilm formation

The expression of several genes encoding proteins involved in motility and attachment was altered in the PAOSX strain compared to the H103 strain (Table 2). These included *pilA*, encoding the pilin subunit PilA of the Type IV pilus, and *flp* that encodes the Type IVb pilin, which were markedly down-regulated (49 and 17 fold, respectively) in the PAOSX mutant. These results were confirmed by qRT-PCR (Table 1). Genes belonging to the *tad* operon encoding the Tad machinery that assembles Type IVb pilin, and the *cupE* operon encoding a non-archetypal chaperon-usher system responsible for assembling cell surface-associated fimbrial structures, were down-regulated in the *sigX* mutant (ranging from -2 to -4.2 fold and -1.9 to -4.4 fold, respectively). Genes belonging to the *bap* operon (PA1874-1877) encoding the externalized BapA adhesin and the ATP-binding cassette (ABC) type I secretion system transporter, were downregulated in the *sigX* mutant (-2.2 to -4.5 fold). We also observed down-regulation in the *sigX* mutant (by -2.2 fold, Table 2) of PprB, a result that was confirmed by qRT-PCR (Table 1, -2.2 fold change). PprB is the response regulator of the two component system PprA/B, which directly and positively controls the expression of three molecular systems involved in biofilm formation, namely the Type IVb Flp pili [27], the chaperone-usher system CupE fimbriae [28] and the BapA adhesin [29]. 

**Figure 2:**
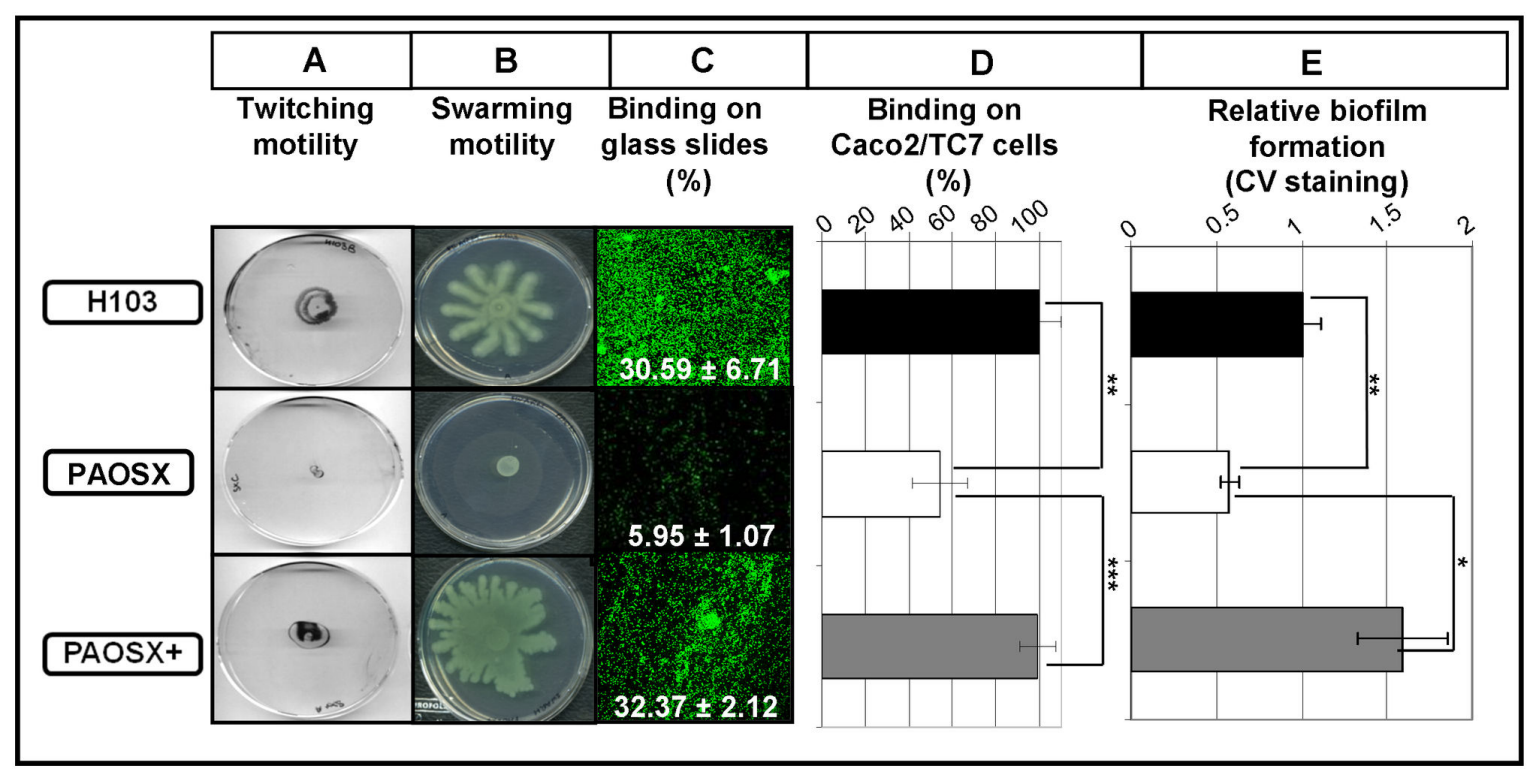
Involvement of SigX in (A) twitching and (B) swarming motilities, attachment to (C) glass slides and to (D) Caco2/TC7 cells, and (E) biofilm formation. Twitching motility was assayed on solidified M9G medium containing 1% agar. Swarming motility was assayed on M9G containing 1% casamino acids as nitrogen source and solidified with 0.5 % agar. For adherence onto glass slides, mid-log phase cultures of GFP expressing bacteria were diluted in 0.9% NaCl to an OD_580_ of 0.6 and allowed to adhere for 2h. Attached cells were observed using a confocal laser scanning microscope and a binding index was calculated (value on each slide). Binding of bacteria onto Caco2/TC7 cells: each bar represents the mean number of adherent bacteria per cell (±SD) calculated by direct microscopic counting of 100 cells and expressed as a percentage compared to the binding of the wildtype H103 strain. For biofilm assay, bacteria were allowed to form a pellicle for 24h at 37°C. Biofilms were quantified by measuring the absorbance at 595 nm after crystal violet (CV) staining. Relative biofilm formation was determined by comparison to the wildtype strain (±SD). Each experiment was performed at least three times. Statistics were done by pairwise strain comparisons (*t* test). *p-value<0.05, **p-value<0.01, ***p-value<0.001.

Type IV pili are required for twitching motility and swarming motility [30]. Consistent with the array data, the *sigX* mutant was highly deficient in these two motilities, that were restored by complementing the mutant with the *sigX* gene (PAOSX+ strain) (Figure 2A and 2B). Type IV and Type IVb pili, fimbriae and BapA adhesin are adhesive organelles that play key roles in the attachment of *P. aeruginosa* to biotic and abiotic surfaces and in cell clustering and biofilm structuring [29,31]. Accordingly, the binding index of PAOSX was reduced on both glass slides (Figure 2C, about 5 fold) and Caco2/TC7 human cells (Figure 2D, 1.8 fold), and the adherence was fully restored in both cases by complementation. Adherence is also involved in biofilm formation that was assayed by measuring the formation of pellicle, a biofilm forming at the air-liquid interface (Figure 2E). Abiotic biofilm formation was reduced by approximately 40% in PAOSX (Figure 2E), which is consistent with the substantial alterations in pilus production. Thus SigX positively regulates multiple pilus/fimbriae/adhesin-dependent processes relevant to acute and chronic colonization and virulence, including swarming and twitching motilities, adherence to abiotic and biotic surfaces, and biofilm formation. 

### Antimicrobial and multidrug resistance and susceptibility

Expression of the *mexXY* gene cluster encoding components of the resistance-nodulation-division (RND)-type efflux systems MexXY/OprM [32] was strongly up-regulated in the PAOSX strain (6 to 13 fold, Table 2), a result that was confirmed by qRT-PCR (see Table 1). The *mexZ* gene encoding the TetR-like repressor of the *mexXY* operon was 5-fold up-regulated, indicating that the *mexXY* dysregulation was independent of MexZ. This might rather result from the up-regulation of the *prfH*-PA5471 gene cluster (6 to 7 fold), the products of which modulate the activity of MexZ [33]. Since the MexXY/OprM efflux pump is involved in intrinsic and mutational aminoglycoside resistance, we assayed MICs of various target antibiotics in PAOSX and H103 strains. As shown in Table 3, PAOSX was only slightly more resistant to aminoglycosides kanamycin and gentamicin and to the fluoroquinolone norfloxacin, but 4 to 30-fold more resistant to tetracycline, nalidixic acid and erythromycin, which could be at least partly attributed to MexXY/OprM efflux pump activity. Finally, the expression of the gene encoding the transcriptional regulator PsrA was decreased (Table 2). Although PsrA is linked to susceptibility to the polycationic polymyxin B [34], the two strains display the same MIC to polymyxin B (Table 3). PsrA also regulates positively the transcription of *rpoS*, encoding the main stationary phase sigma factor [34,35]. Although many genes belonging to the RpoS regulon were down-regulated in PAOSX (Table 2), e. g. *psrA* [35], suggesting that RpoS is less active than in the wildtype strain, the transcription of *rpoS* itself was not significantly altered in PAOSX strain under our growth conditions (data not shown). 

**Table 3:** MICs to antibiotics.

**Class**	**Antibiotic**	**H103 (µg/mL)**	**PAOSX (µg/mL)**
Polymyxin	Polymyxin B	1	1
Tetracycline	Tetracycline	15.6	500
Quinolones	Nalidixic acid	31.25	125
	Norafloxacin	0.5	1
Aminoglycosides	Kanamycin	400	800
	Gentamicin	2	4
Macrolides	Erythromycin	125	500

 The *asrA* gene (PA0779), encoding an aminoglycoside-induced stress response ATP-dependent protease [36] was up-regulated by 2.4 fold (Table 2). Mutants in *asrA* are aminoglycoside super-susceptible, and *asrA* overexpression leads to an increase of most heat shock and chaperone genes transcription [36]. Accordingly, our microarray data showed that several of these chaperone genes (*hptG, groESL, clpB, dnaK* and *hslV*) were up-regulated in the *sigX* mutant by 2 to 4.4 fold (Table 2). To confirm that these expression data are functionally relevant, bacteria were subjected to heat shock stress. The PAOSX mutant was about 3-fold more sensitive to heat treatment than either the wildtype H103 or the complemented mutant strains (Figure S2A), indicating that SigX assists in protection against heat shock. 

### The *sigX* mutant is affected in secretion systems and expression of secreted factors

**Figure 3:**
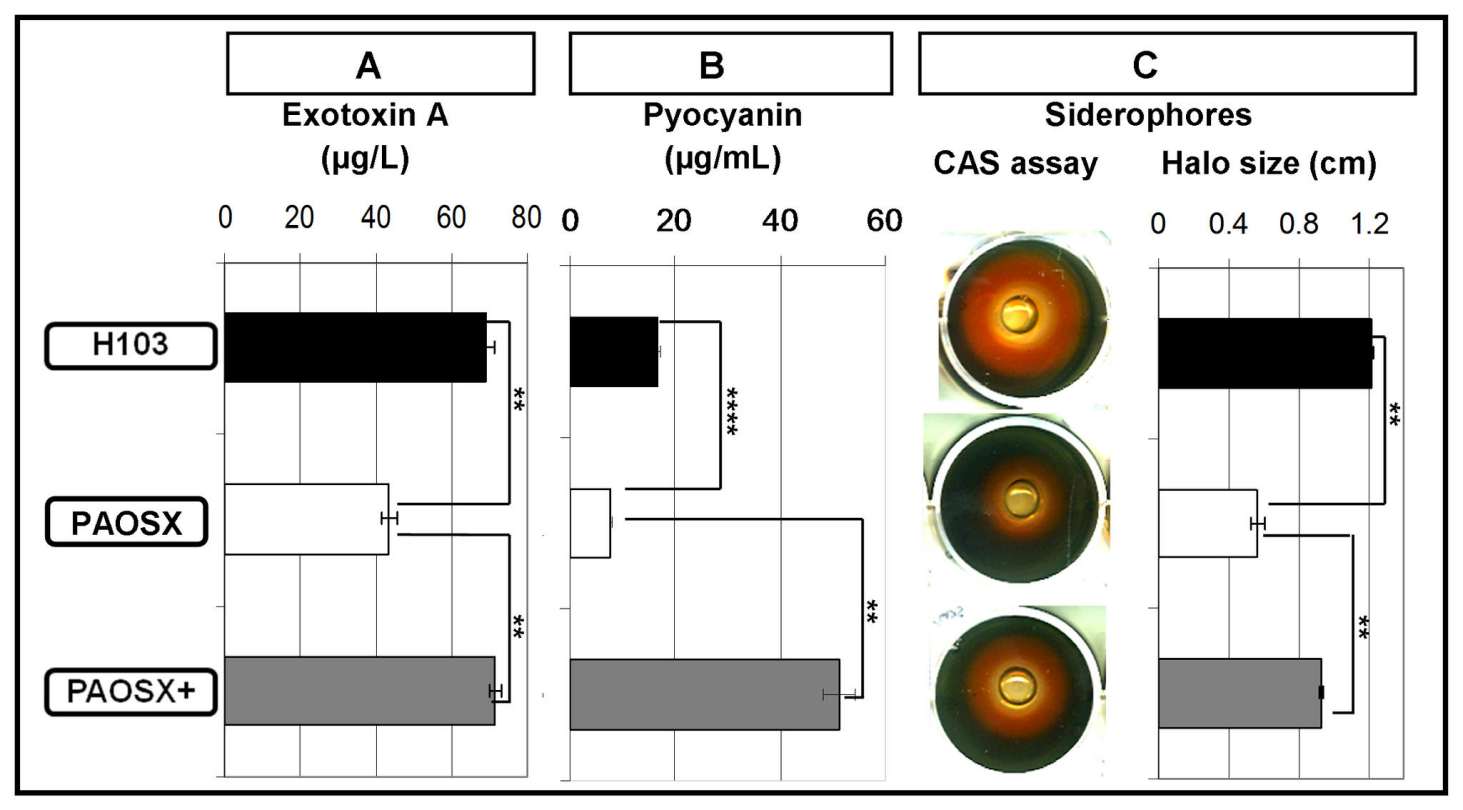
Altered levels of (A) exotoxinA, (B) pyocyanin and (C) siderophores in the culture supernatants of the *sigX* mutant. H103 (black), PAOSX (white) and PAOSX+ (grey) culture supernatants were obtained from overnight cultures in (A) LB, (B) King A or (C) King B media. The relative amounts of exotoxin A, pyocyanin and the total siderophores were assayed at least three times independently for each strain and means and standard deviations are presented. For the Chrome Azurol S (CAS) assay, the haloes around the wells in the CAS plate show siderophore production in sample supernatant. Statistics were done by pairwise strain comparisons (*t* test). *p-value<0.05, **p-value<0.01, ***p-value<0.001, **** *p*-value<0.0001, NS no significant difference.

We observed the dysregulation of genes involved in secretion systems and/or in their effectors. Genes related to the Type 1 secretion systems (Apr, Has and Bap), the Xcp Type 2 secretion system, and the HsiIII Type 6 secretion system were down-regulated in PAOSX strain, while genes related to the Type 3 and HsiI Type 6 secretion systems were up-regulated (Table 2). With regards to effectors, *toxA*, which encodes the exotoxin A secreted by the Xcp Type II secretion system, was 5 fold down-regulated in the *sigX* mutant strain. Accordingly, production of exotoxin A was 1.6 fold reduced in culture supernatants of PAOSX compared to both the wild type strain and the *sigX*-complemented mutant PAOSX+ strain (Figure 3A). The expression of other genes that synthesize secreted factors were up-regulated in the *sigX* mutant. This was the case for the *hcnABC* genes which were between 2.5 and 5.6 fold up-regulated in PAOSX strain, as well as PA3912-PA3913 (2.3 and 4 fold) encoding two probable proteases with homologies to collagenases (http://www.pseudomonas.com, [18]). The *rhlA-rhlB* genes encoding key enzymes for rhamnolipid synthesis [37], and several genes (*phzD2-G2, phzS*) involved in pyocyanin production [38] were also down-regulated between 3.3 and 7.8 fold (Table 2). While rhamnolipid production was not substantially altered in the PAOSX mutant (data not shown), pyocyanin production was reduced approximately two fold in the PAOSX culture supernatants compared to those of H103. Furthermore, the complemented mutant over-produced this phenazine by 3.5 fold compared to the wildtype strain (Figure 3B), likely due to the higher expression of SigX in the complemented strain. Taken together, our data demonstrated a dysregulation of many genes encoding secretion system components and effectors in the *sigX* mutant. 

### Effect of the *sigX* mutation on transcription of iron uptake systems

One of the most important mechanisms of acquisition of the essential nutrient iron is the secretion of iron-chelating siderophores under iron-depleted conditions. These molecules bind ferric iron with high affinity and are taken up by dedicated pathways involving specific outer membrane receptors [39]. Several siderophore synthesis and siderophore receptor genes were down-regulated in the *sigX* mutant (Table 2). For example, pyochelin biosynthesis requires five operons, of which four displayed down-regulated expression in the *sigX* mutant strain (namely the *pchDCBA* operon, the *pchEF* genes from the *pchEFGHI* operon, and the PA4220-*fptA* and *ampPO* operons). *P. aeruginosa* can also use the *Vibrio cholerae* siderophore vibriobactin as an iron source, since it produces FvbA, a protein highly homologous to the *V. cholerae* vibriobactin receptor ViuA [40]. The expression of *fbvA* was down-regulated in PAOSX, as was the *viuB*-PA2034 gene cluster, encoding the cytoplasmic ViuB that catalyses the removal of iron from the ferri-vibriobactin complex. To extend these expression data, we assayed total siderophore production using chrome azurol S (CAS) agar plates. As shown on Figure 3C, the production of siderophores was around 2-fold reduced in the *sigX* mutant strain, compared to the wildtype and complemented PAOSX+ strains. 

We also observed the down-regulation of PA3899, encoding an ECF sigma factor that is part of a cell surface signaling system for iron citrate uptake [5], and PA1301, involved in the activity of the ECF sigma factor PA1300 that regulates haem uptake system [5]. *P. aeruginosa* can also directly use haem produced by eukaryotes as an iron source, through the Has and Phu systems [41]. In PAOSX, the haem-acquisition *has* genes (*hasRADEF*) were strongly down-regulated (Table 2). We also observed the down-regulation of the PA0781 gene encoding a putative TonB-dependent receptor family protein homologous to the outer membrane receptor PhuR, which might be of importance under iron starvation encountered in the eukaryotic host [18]. Finally, many genes that were dysregulated in the *sigX* mutant and involved in iron homeostasis, are commonly repressed by the ferric uptake regulator Fur (as indicated in Table 2), indicating that Fur was more active in the *sigX* mutant, although the *fur* gene itself was not significantly dysregulated under our conditions (data not shown).

### Effect of the *sigX* mutation on expression of genes involved in energy metabolism

Several genes encoding products related to energy metabolism were differentially expressed between PAOSX and H103 strains, including many cytochromes and ubiquinol oxidases (Table 2) that are involved in electron transfer pathways during aerobic respiration. For example, the *cox* (PA0105-PA108) and the *cyo* (PA1317-PA1320) operons, encoding the Aa3 cytochrome c oxidase and the Bo_3_ quinol oxidase, respectively, which play a dominant role under high oxygen conditions [42,43], were between 2.7 and 4.1 fold down-regulated in the *sigX* mutant. In contrast, the *ccoNOPQ-2* gene cluster (PA1555-PA1557) encoding the cytochrome Cbb3-2 oxidase, which is up-regulated under low oxygen conditions [42,43], was up-regulated by 4.6 and 6.7 fold in the PAOSX mutant compared to H103 wildtype strain (Table 2). These data suggest that the expression of genes involved in the aerobic respiration pathway was reduced in the absence of SigX. Conversely, several genes that specify products involved in denitrification were up-regulated in the PAOSX mutant compared to H103 strain, including the *nirSMCFD* genes (PA0515-PA0519; an average of ~8 fold, Table 2), *anr* (1.6 fold), *narL* (4.7 fold), and the operon *dnr-PA0526* (3.8 and 5 fold), which encode the three major transcriptional regulators of anaerobiosis, N-oxide-sensing response and dissimilatory nitrate respiration pathways, respectively (Table 2) [44,45]. The microarray data corresponding to these three regulators were confirmed by qRT-PCR (Table 1). There was also an increased expression of the gene cluster PA1196-PA1197 (between 4 to 6 fold, Table 2), encoding a putative transcriptional regulator involved in nitrogen utilization genes, and of *uspL*, *uspM*, *uspO* and *PA4352* (between 2.3 and 4 fold, Table 2), encoding universal stress response proteins. Some of these genes are known to be activated by the global anaerobiosis regulator Anr [45] that was marginally upregulated by 1.6 fold (Table 1), which might indicate that Anr is more active in PAOSX than in H103. Finally, when bacteria were grown to mid-log phase and subjected to oxygen depletion, the growth of the wildtype strain was reduced, while that of the PAOSX *sigX* mutant was less affected, suggesting that PAOSX could be better adapted for growing under these conditions than H103 (Figure S2B).

### PAOSX mutant shows reduced virulence

**Figure 4:**
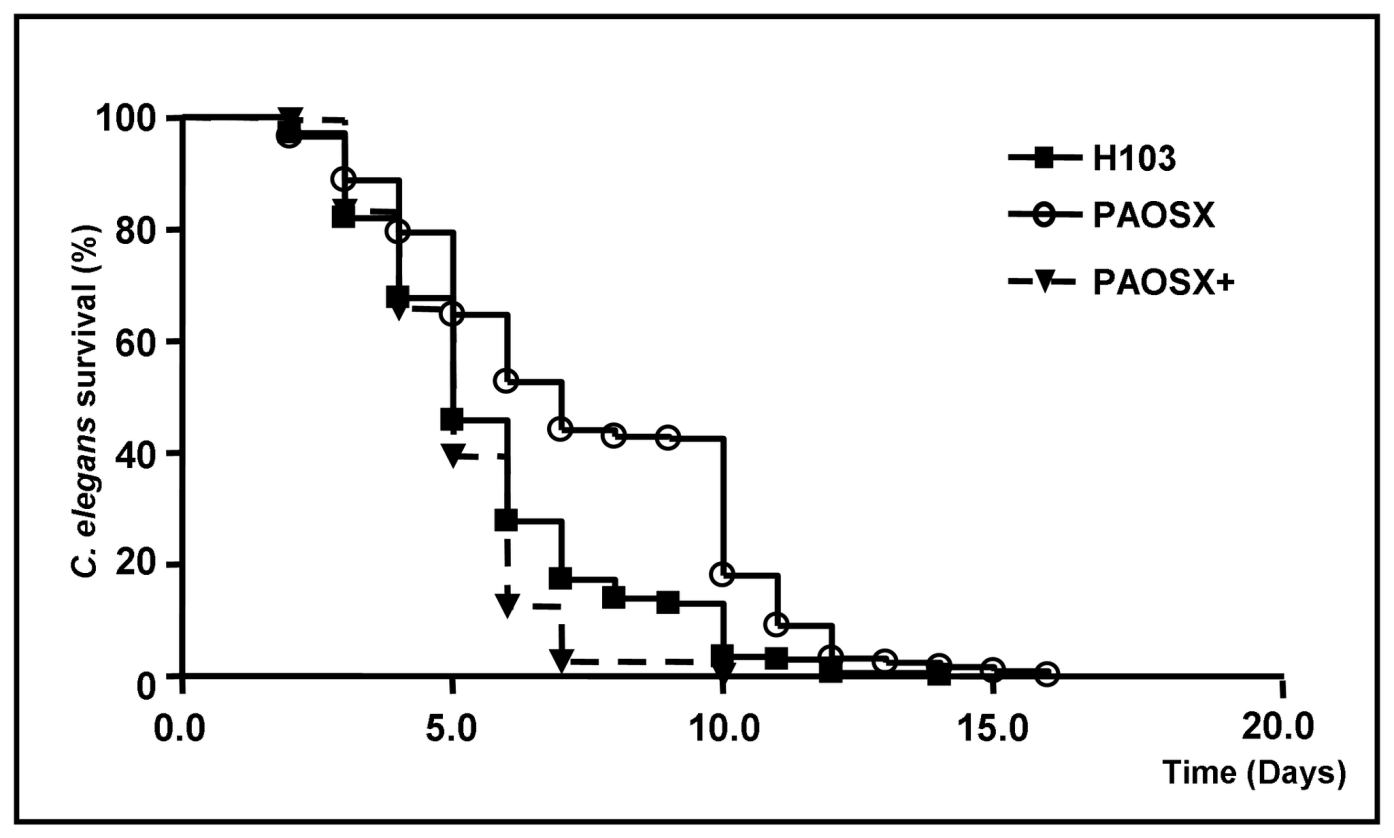
The absence of SigX modulated *P*. *aeruginosa* virulence in the *C*. *elegans* model. Kaplan-Meier survival plots of *C*. *elegans* nematodes fed with the wild type strain *P*. *aeruginosa* H103 (n = 210), the *sigX* mutant PAOSX (n = 257), and the SigX-complemented mutant strain PAOSX+ (n = 201). Each value reported for the assay is the mean of measurements of eight samples from three independent experiments. Pairwise strain comparisons (log rank test) were as follows: H103 *versus* PAOSX, *p*-value< 0.0001; PAOSX *versus* PAOSX+, *p*-value< 0.0001; H103 *versus* PAOSX+, *p*-value <0.001. Four independent experiments were performed.

The virulence of the three strains was assessed using the nematode *Caenorabditis elegans*. *P. aeruginosa* is able to kill *C. elegans* in an infection-like process that requires the ingestion of bacteria, followed by proliferation in the worm gut [46]. As shown in Figure 4, *P. aeruginosa* H103 wildtype and PAOSX+ were very toxic to the worms since about 50-60% of the population died within 5 days. In contrast, the virulence of the *sigX* mutant PAOSX was reduced since it required 7 days of exposure to kill approximately 50% of the worms. At 10 days postinfection, near 100% of the worms exposed to H103 and PAOSX+ were dead, whereas around 18% of the initial worm population was still alive on plates seeded with PAOSX. Pairwise strain comparisons led to the conclusion that the survival curve for the mutant was significantly different from those of the wildtype strain and of the complemented mutant, indicating that SigX is involved in *P. aeruginosa* virulence in this model (Figure 4). 

## Discussion

Apart from its role in transcription of *oprF*, which encodes the major outer membrane porin [6,7], no function had been previously assigned to SigX, one of the 19 ECF sigma factors of *P. aeruginosa*. Our results indicate that SigX regulates virulence towards a *C. elegans* model as well as virulence-related behaviors in *P. aeruginosa*. Since the growth of PAOSX was altered in LB rich medium, and since slow killing of *C. elegans* is correlated with the proliferation of live bacteria in the worm gut [47], the reduced virulence of PAOSX against *C. elegans* could be related, at least partly, to its putative lower generation time in the worm gut. The death kinetics of *C. elegans* may also be due primarily to the interaction between worms and the bacteria during the first hours of contact [48], suggesting that other factors than growth alterations may be affected in the mutant strain. These data show an obligate role for SigX in swarming and twitching motilities, as well as an involvement in adhesion to abiotic and biotic surfaces, and in biofilm formation. These results are consistent with a substantial downregulation of the transcription of Type IV pili. We observed in the *sigX* mutant altered expression of *pprB*, the regulator of the two-component system PprA/B, which positively regulates the expression of adhesive organelles (BapA, CupE and type IVb pili), and thus plays a role in cell clustering and biofilm structuring [28,29]. We also observed altered antibiotic resistance phenotypes that could be explained in part by the over-expression of the efflux pump MexXY/OprM, a key element of bacterial adaptation to antibiotics targeting the ribosome [49], coupled to the reduced expression of the major porin OprF that has been proposed to be involved in antibiotic uptake in *P. aeruginosa* [50]. Interestingly, PprB-dependent repression of the *mexXY* operon has been recently shown in *P. aeruginosa* [29], suggesting that the over-expression of *mexXY* in the *sigX* mutant could be linked, at least partly, to the altered expression of *pprB* in the *sigX* mutant. 

SigX also affected the transcription of six different secretion systems. In addition, its modulation of iron and haem capture and scavenging systems is consistent with a role in iron sequestration, which is essential for *in vivo* survival and dissemination. Several virulence factors were also affected in PAOSX, which may reflect the postulated modulation of the Anr and Fur-Fe^2+^ regulators in the *sigX* mutant. In previous studies, we showed that *P. aeruginosa* requires OprF for full virulence [11] and for rhamnolipid production [51]. However, *oprF* is only around 1.5 (by arrays, Table 1) or 2.2 (by qRT-PCR, Table 1) fold down-regulated in PAOSX and the *oprF* mutant does not display many of the PAOSX phenotypes described here (this study and [7]). Taken together, these data clearly indicate that OprF cannot account for all of the phenotypes observed in the *sigX* mutant. The link between an ECF sigma factor and virulence was previously demonstrated in several bacterial genera, including *Porphyromonas gingivalis* [52], *Staphylococcus aureus* [53], and *Mycobacterium tuberculosis* [54]. In *P. aeruginosa* the cell surface signaling system PUMA3 was shown to positively control the *hxc* alternative Type II secretion system [26]. The strong down-regulation in the *sigX* mutant of genes (*hasAP*, PA0781, PA3600/PA3601) that have been recently shown to be over-expressed during chronic biofilm-associated infections, and that are important for *P. aeruginosa* virulence [55] may support the link between SigX and biofilm formation and virulence. 

The Anr and Dnr proteins are two global transcriptional regulators homologous to Fnr (fumarate nitrate reductase regulator), and involved in anaerobiosis [56] and response to nitrogen oxides [57], respectively. Here we observed that *anr* was moderately up-regulated (1.6 fold) while *dnr* was strongly up-regulated (3.8 to 5.8 fold) in the *sigX* mutant (Table 2). Consistently, several Anr/Dnr-regulated genes were up-regulated (see Table 2, Anr-regulated genes). Thus it seems likely that Anr and Dnr are more active in the *sigX* mutant than in the wildtype strain. Overall, since our mutant was grown under largely aerobic conditions, rather than the anaerobic conditions normally required to activate the Anr and Dnr regulators, these data may suggest a role for SigX in suppressing the production of genes required for the anaerobic lifestyle. Interestingly in *P. gingivalis*, the ECF sigma factor SigH is upregulated by exposure to molecular oxygen, suggesting a role in adaptation of this bacterium to oxygen [58]. 

In general, the most studied ECF sigma factors belong to the categories of stress-responsive (RpoE-like) and iron starvation (FecI-like) sigma factors [2]. The first ones respond to stress/cell envelope damage and regulate genes that restore proper function of the cell in these conditions. FecI-like sigma factors normally respond to the presence of a specific siderophore and regulate iron uptake. Activity of this group of sigma factors is mainly controlled by cell-surface signalling in Gram negative bacteria, while this regulatory system does not control the activity of RpoE-like sigma factors. In *P. aeruginosa*, many ECF sigma factors are involved in iron uptake and/or iron homeostasis, but usually tend to be involved in regulating the uptake of specific siderophore-iron complexes [4,5]. SigX has functions partly overlapping those of these ECF sigma factors, since the *sigX* mutant displays modest changes in expression of genes involved in multiple siderophore, iron citrate and haem uptake systems as well as moderate reductions in siderophore production. Unlike the other ECF sigma factors involved in metal (mostly iron) uptake [5,59], SigX appears relatively non-specific and lacks other elements of cell surface signalling systems (anti-sigma factor and TonB-dependent transducer) encoded by genes in the vicinity of the *sigX* gene. Furthermore, ECF sigma factors that belong to cell surface signaling systems (CSS) are known to regulate limited numbers of genes, most of them being located in the vicinity of the gene encoding the ECF sigma factor [5,26], while stress-responsive ECF sigma factors regulate expression of many genes. Our microarray analysis showed that SigX modulates the expression of 307 genes (~8% of the *P. aeruginosa* genome) by more than 2-fold although many of these genes might be regulated in an indirect manner, indicating that SigX was active under the growth conditions utilized. The activity of most ECF sigma factors is post-transcriptionnaly controlled by an anti-sigma factor, and activated in response to an environmental signal. Since *sigX* is not clustered and co-transcribed with an anti-sigma factor [18], it is difficult to predict its mechanism of activation. It is noticeable that not all ECF sigma factors are linked to potential anti-sigma factors, suggesting the existence of alternative pathways in the control of sigma factor activity [2]. In some cases the ECF sigma factor might be regulated only at the transcriptional level, as has been described for *Streptomyces coelicolor* SigE, which is regulated by the CseBC two-component system in response to cell envelope stress [60]. 

The high number of genes whose expression was affected by the sig*X* mutation would place SigX as a putative pleiotropic regulator in *P. aeruginosa*, at a level similar to that of the well-known *P. aeruginosa* AlgU ECF sigma factor [61], the cell envelope stress regulon of which is composed of 293 genes [62]. Finally, RpoE-like sigma factors usually control their own expression, like SigX, which is not the case of FecI-like sigma factors. Taken together, the features of SigX make it more closely related to stress-responsive than to FecI-like sigma factors. This hypothesis is further strengthened by i) the overexpression of *sigX* in cell wall stress conditions, induced by D-cycloserine [62], or in low shear modelled microgravity [63], and ii) by the high sequence similarities with the well-studied SigW ECF sigma factor of *Bacillus subtilis* [64] that is induced by alkaline shock, phage infection, salt and antibiotics affecting cell-wall biosynthesis [12,13].

Overall our study is consistent with the hypothesis that master regulators are affected in the *sigX* mutant in terms of expression and/or activity since we noted at least 9 dysregulated regulators, four of which, namely *sigX* itself, *psrA*, PA3899 and *pprB*, were down-regulated. This may suggests that SigX sits atop of a regulatory hierarchy leading to the regulation of a large panel of genes, most of them probably in an indirect manner. It will be of interest in future studies to identify the direct targets of the ECF sigma factor. Since the absence of SigX leads to attenuated bacterial virulence and biofilm formation, and altered antibiotic or drug resistance/susceptibility, understanding the mechanisms by which *sigX* is expressed and activated, will constitute a significant challenge that could potentially shed light on the complex regulation of pathogenesis in *Pseudomonas*. 

## Supporting Information

Figure S1 **Growth kinetics of the wildtype strain H103 (black squares), its *sigX* deficient mutant PAOSX (open squares) and the *sigX*-complemented PAOSX strain (grey squares) in LB (**A**) or M9G (**B**) medium.** Experiments were repeated at least three times.
(TIF)

Figure S2 **Resistance of the PAOSX mutant strain to heat shock (A) or oxygen depleted medium (B) treatments.** Bacteria were grown to mid-log phase in M9G medium at 37°C. For heat shock assays, bacteria were diluted at 10^7^ CFU.ml ^-1^ before being shocked at 50°C for 10 min or not. Cells were then plated on solidified LB medium, and allowed to grow for 24h at 37°C before being numerated. Results are given as the percentage of the ratio: CFU after heat treatment/CFU without heat treatment. For anaerobiosis assays, 50 mM NaNO_3_ was added to the culture, which was covered with a thick layer of mineral oil (indicated by an arrow). The growth kinetic was followed. Statistics were done by pairwise strain comparisons (*t* test). *p-value<0.05 Experiments were repeated at least three times.
(TIF)

Table S1 **List of genes differentially expressed in PAOSX mutant in M9G medium (log_2_ ≥ 2) with a *p*-value ≤ 0.05.**
(DOC)

Table S2 **Primer sequences of the indicated genes used for quantitative RT-PCR reactions.**
(DOC)
