# Supplementary figures and images for: The Extra-Cytoplasmic Function Sigma Factor SigX Modulates Biofilm and Virulence-Related Properties in Pseudomonas aeruginosa
Source: PLoS One. 2013 Nov 18;8(11):e80407. doi: 10.1371/journal.pone.0080407 (PMC3832394; doi:10.1371/journal.pone.0080407)

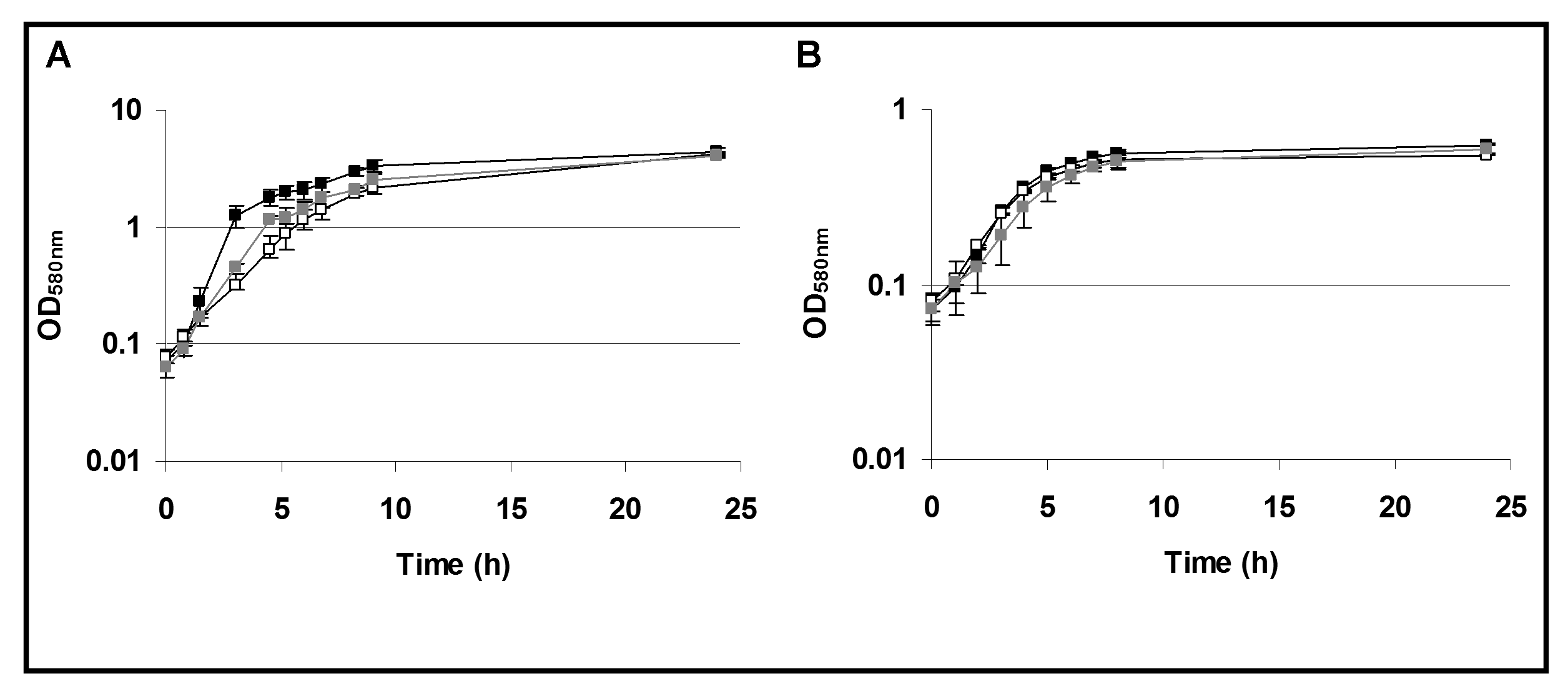

Supplement: Figure S1 — Growth kinetics of the wildtype strain H103 (black squares), its sigX deficient mutant PAOSX (open squares) and the sigX-complemented PAOSX strain (grey squares) in LB (A) or M9G (B) medium. Experiments were repeated at least three times. (TIF) [file pone.0080407.s001.tif]

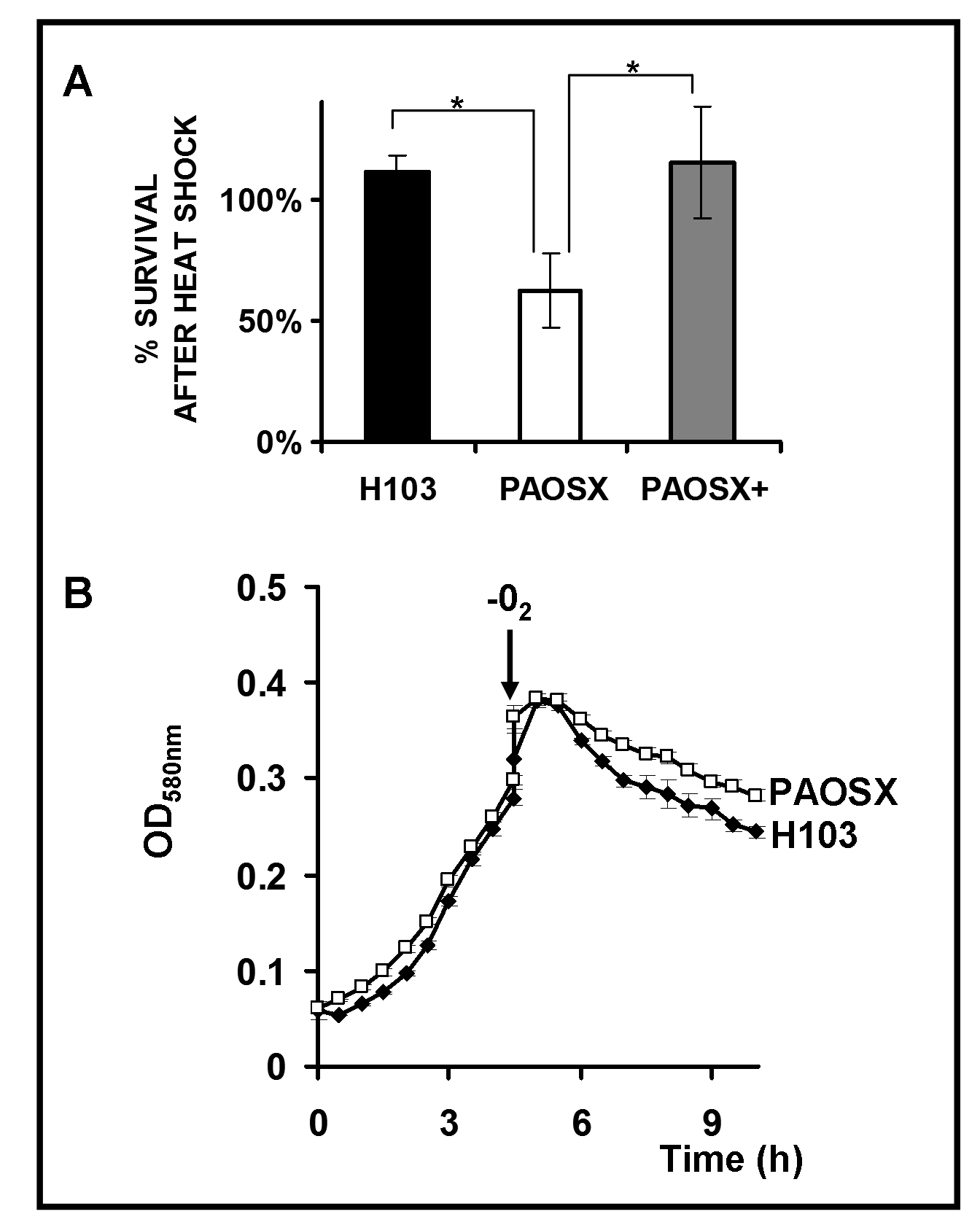

Supplement: Figure S2 — Resistance of the PAOSX mutant strain to heat shock (A) or oxygen depleted medium (B) treatments. Bacteria were grown to mid-log phase in M9G medium at 37°C. For heat shock assays, bacteria were diluted at 107 CFU.ml -1 before being shocked at 50°C for 10 min or not. Cells were then plated on solidified LB medium, and allowed to grow for 24h at 37°C before being numerated. Results are given as the percentage of the ratio: CFU after heat treatment/CFU without heat treatment. For anaerobiosis assays, 50 mM NaNO3 was added to the culture, which was covered with a thick layer of mineral oil (indicated by an arrow). The growth kinetic was followed. Statistics were done by pairwise strain comparisons (t test). *p-value<0.05 Experiments were repeated at least three times. (TIF) [file pone.0080407.s002.tif]
