# Supplementary material for: The Extra-Cytoplasmic Function Sigma Factor SigX Modulates Biofilm and Virulence-Related Properties in Pseudomonas aeruginosa
Source: PLoS One. 2013 Nov 18;8(11):e80407. doi: 10.1371/journal.pone.0080407 (PMC3832394; doi:10.1371/journal.pone.0080407)
Supplement: Table S1 — List of genes differentially expressed in PAOSX mutant in M9G medium (log2 ≥ 2) with a p-value ≤ 0.05. (DOC) [file pone.0080407.s003.doc]

**Supplementary Table S1: list of genes differentially expressed in PAOSX mutant in M9G with a *p*-value ≤ 0.05 and a fold change of at least 2**.

| **Gene**  **number** | **Gene Symbol** | **Product name and/or function** | **Fold change (log_2_)** |
| --- | --- | --- | --- |
| PA0024 | *hemF* | Coproporphyrinogen III oxidase | 4.2 |
| PA0039 | *---* | Hypothetical protein | -2.2 |
| PA0044 | *exoT* | Exoenzyme T | 3.9 |
| PA0080 | *tssJ1* | Type VI secretion system protein TssJ1 | 2 |
| PA0083 | *tssB1* | Type VI secretion system protein TssB1 | 2.9 |
| PA0084 | *tssC1* | Type VI secretion system protein TssC1 | 2.3 |
| PA0085 | *hcp1* | Type VI protein secretion system component Hcp | 2.5 |
| PA0086 | *tagJ1* | Type VI secretion system protein TagJ1 | 2.3 |
| PA0090 | *clpV1* | ClpV1 | 2.3 |
| PA0105 | *coxB* | Cytochrome c oxidase. subunit II | -4.1 |
| PA0106 | *coxA* | Cytochrome c oxidase. subunit I | -4 |
| PA0107 | *---* | Hypothetical protein | -3 |
| PA0108 | *coIII* | Cytochrome c oxidase. subunit III | -3 |
| PA0110 | *---* | Hypothetical protein | -3 |
| PA0111 | *---* | Hypothetical protein | -3 |
| PA0122 | *rahU* | RahU | -2.9 |
| PA0126 | *---* | Hypothetical protein | 2.5 |
| PA0208 | *mdcA* | Malonate decarboxylase alpha subunit | 4.1 |
| PA0209 | *---* | Hypothetical protein | 3.4 |
| PA0210 | *mdcC* | Malonate decarboxylase delta subunit | 3 |
| PA0211 | *mdcD* | Malonate decarboxylase beta subunit | 2.4 |
| PA0213 | *---* | Hypothetical protein | 2.7 |
| PA0214 | *---* | Probable acyl transferase | 2.3 |
| PA0271 | *---* | Probable acyl transferase | -2.5 |
| PA0294 | *aguR* | Transcriptional regulator AguR | 2 |
| PA0295 | *---* | Probable periplasmic polyamine binding protein | 4.1 |
| PA0315 | *---* | Hypothetical protein | -3.3 |
| PA0403 | *pyrR* | Transcriptional regulator PyrR | 2.8 |
| PA0408 | *pilG* | Twitching motility protein PilG | 2.8 |
| PA0409 | *pilH* | Twitching motility protein PilH | 2.5 |
| PA0410 | *pilI* | Twitching motility protein PilI | 2.3 |
| PA0423 | *pasP* | PasP | -2 |
| PA0506 | *---* | Probable acyl-CoA dehydrogenase | -3.2 |
| PA0515 | *---* | Probable transcriptional regulator | 7.1 |
| PA0516 | *nirF* | Heme d1 biosynthesis protein NirF | 3.1 |
| PA0517 | *nirC* | Probable c-type cytochrome precursor | 8.1 |
| PA0518 | *nirM* | Cytochrome c-551 precursor | 8.6 |
| PA0519 | *nirS* | Nitrite reductase precursor | 13.1 |
| PA0526 | *---* | Hypothetical protein | 5 |
| PA0527 | *dnr* | Transcriptional regulator Dnr | 3.9 |
| PA0542 | *---* | Hypothetical protein | 3.3 |
| PA0572 | *---* | Hypothetical protein | -4.1 |
| PA0586 | *---* | Hypothetical protein | -3.5 |
| PA0587 | *---* | Hypothetical protein | -3.4 |
| PA0588 | *---* | Hypothetical protein | -2.2 |
| PA0656 | *---* | Probable HIT family protein | -2.2 |
| PA0665 | *---* | Hypothetical protein | -2.9 |
| PA0713 | *---* | Hypothetical protein | 2.2 |
| PA0731 | *---* | Hypothetical protein | 2.3 |
| PA0743 | *---* | Probable 3-hydroxyisobutyrate dehydrogenase | -2.2 |
| PA0754 | *---* | Hypothetical protein | 2.7 |
| PA0755 | *opdH* | Cis-aconitate porin OpdH | 2.2 |
| PA0779 | *asrA* | AsrA | 2.5 |
| PA0781 | *---* | Hypothetical protein | -7.7 |
| PA0787 | *---* | Hypothetical protein | 2.4 |
| PA0792 | *prpD* | Propionate catabolic protein PrpD | -3.3 |
| PA0836 | *ackA* | Acetate kinase | 3 |
| PA0852 | *cbpD* | Chitin-binding protein CbpD precursor | -2.9 |
| PA0918 | *---* | Cytochrome b561 | 2.5 |
| PA0958 | *oprD* | Basic amino acid outer membrane porin | -2.9 |
| PA0959 | *---* | Hypothetical protein | -2 |
| PA0960 | *---* | Hypothetical protein | -2.3 |
| PA1041 | *---* | Probable outer membrane protein precursor | -2.4 |
| PA1049 | *pdxH* | Pyridoxine 5'-phosphate oxidase | 3.8 |
| PA1050 | *---* | Hypothetical protein | 2.3 |
| PA1053 | *---* | Hypothetical protein | -2.5 |
| PA1076 | *---* | Hypothetical protein | 3 |
| PA1100 | *fliE* | Flagellar hook-basal body complex protein FliE | 2.3 |
| PA1123 | *---* | Hypothetical protein | 2.5 |
| PA1127 | *---* | Probable oxidoreductase | -2.5 |
| PA1148 | *toxA* | Exotoxin A precursor | -5.1 |
| PA1190 | *---* | Hypothetical protein | -3.3 |
| PA1196 | *---* | Probable transcriptional regulator | 6 |
| PA1197 | *---* | Hypothetical protein | 4.1 |
| PA1244 | *---* | Hypothetical protein | -2.1 |
| PA1245 | *aprX* | AprX | -2.9 |
| PA1246 | *aprD* | Alkaline protease secretion protein | -2 |
| PA1249 | *aprA* | Alkaline metalloproteinase precursor | -3.1 |
| PA1289 | *---* | Hypothetical protein | -3 |
| PA1301 | *---* | Probable transmembrane sensor | -2.7 |
| PA1317 | *cyoA* | Cytochrome o ubiquinol oxidase subunit II | -2.8 |
| PA1318 | *cyoB* | Cytochrome o ubiquinol oxidase subunit I | -2.8 |
| PA1319 | *cyoC* | Cytochrome o ubiquinol oxidase subunit III | -3 |
| PA1320 | *cyoD* | Cytochrome o ubiquinol oxidase subunit IV | -3 |
| PA1429 | *---* | Probable cation-transporting P-type ATPase | 4.2 |
| PA1455 | *fliA* | Sigma factor FliA | 2.1 |
| PA1546 | *hemN* | Oxygen-independent coproporphyrinogen III oxidase | 3.1 |
| PA1550 | *---* | Hypothetical protein | 2.6 |
| PA1551 | *---* | Probable ferredoxin | 2.9 |
| PA1555 | *ccoP2* | Cytochrome c oxidase, cbb3-type, CcoP subunit | 6.1 |
| PA1556 | *ccoO2* | Cytochrome c oxidase, cbb3-type, CcoO subunit | 6.7 |
| PA1557 | *ccoN2* | Cytochrome c oxidase, cbb3-type, CcoN subunit | 4.6 |
| PA1596 | *htpG* | Heat shock protein HtpG | 3.5 |
| PA1597 | *---* | Hypothetical protein | 2.1 |
| PA1604 | *---* | Hypothetical protein | 2.8 |
| PA1647 | *---* | Probable sulfate transporter | 5.2 |
| PA1673 | *---* | Hypothetical protein | 4.9 |
| PA1701 | *pcr3* | Pcr3 | 2.6 |
| PA1706 | *pcrV* | Type III secretion protein PcrV | 3.3 |
| PA1707 | *pcrH* | Regulatory protein PcrH | 3.2 |
| PA1746 | *---* | Hypothetical protein | 5.2 |
| PA1747 | *---* | Hypothetical protein | 2.7 |
| PA1774 | *crfX* | CrfX protein | -2.5 |
| PA1775 | *cmpX* | Conserved cytoplasmic membrane protein, CmpX | -2.9 |
| PA1776 | *sigX* | ECF sigma factor SigX | -4.1 |
| PA1789 | *---* | Hypothetical protein | 3.2 |
| PA1852 | *---* | Hypothetical protein | -7 |
| PA1874 | *---* | Hypothetical protein | -4.4 |
| PA1875 | *---* | Probable outer membrane protein precursor | -2.5 |
| PA1876 | *---* | Probable ATP-binding/permease fusion ABC transporter | -2.4 |
| PA1877 | *---* | Probable secretion protein | -2.2 |
| PA1902 | *phzD1/ phzD2* | Phenazine biosynthesis protein PhzD | -5.4 |
| PA1903 | *phzE1 /phzE2* | Phenazine biosynthesis protein PhzE | -5.5 |
| PA1904 | *phzF1 / phzF2* | Probable phenazine biosynthesis protein | -7.2 |
| PA1905 | *phzG2* | Probable pyridoxamine 5'-phosphate oxidase | -7.8 |
| PA1914 | *hvn* | Halovibrin | -7 |
| PA2018 | *mexY* | RND multidrug efflux transporter | 6.7 |
| PA2019 | *mexX* | RND multidrug efflux transporter | 13.3 |
| PA2020 | *---* | Probable transcriptional regulator | 5.3 |
| PA2023 | *galU* | UTP--glucose-1-phosphate uridylyltransferase | 2.3 |
| PA2024 | *---* | Probable ring-cleaving dioxygenase | -2 |
| PA2033 | *---* | Hypothetical protein | -5.5 |
| PA2034 | *---* | Hypothetical protein | -4.1 |
| PA2067 | *---* | Probable hydrolase | -3.1 |
| PA2068 | *---* | Probable major facilitator superfamily (MFS) transporter | -2.4 |
| PA2069 | *---* | Probable carbamoyl transferase | -3.5 |
| PA2119 | *---* | Alcohol dehydrogenase (Zn-dependent) | 4.5 |
| PA2126 | *cgrC* | CupA gene regulator C, CgrC | 4.8 |
| PA2127 | *cgrA* | CupA gene regulator A, CgrA | 5.9 |
| PA2191 | *exoY* | Adenylate cyclase ExoY | 2.8 |
| PA2193 | *hcnA* | Hydrogen cyanide synthase HcnA | 5.6 |
| PA2194 | *hcnB* | Hydrogen cyanide synthase HcnB | 2.9 |
| PA2195 | *hcnC* | Hydrogen cyanide synthase HcnC | 2.5 |
| PA2223 | *---* | Hypothetical protein | -2.8 |
| PA2290 | *gcd* | Glucose dehydrogenase | -2.2 |
| PA2300 | *chiC* | Chitinase | -3.5 |
| PA2360 | *---* | Hypothetical protein | -2.8 |
| PA2366 | *---* | Uricase PuuD | -2.6 |
| PA2367 | *---* | Hypothetical protein | -3.3 |
| PA2368 | *---* | Hypothetical protein | -3.5 |
| PA2369 | *---* | Hypothetical protein | -2.3 |
| PA2370 | *---* | Hypothetical protein | -4.2 |
| PA2371 | *---* | Probable ClpA/B-type protease | -3.8 |
| PA2373 | *---* | Hypothetical protein | -2.9 |
| PA2377 | *---* | Hypothetical protein | -2.3 |
| PA2434 | *---* | Hypothetical protein | -2.1 |
| PA2442 | *gcvT2* | **G**lycine cleavage system protein T2 | -2.6 |
| PA2443 | *sdaA* | L-serine dehydratase | -2.3 |
| PA2444 | *glyA2* | Serine hydroxymethyltransferase | -2.8 |
| PA2445 | *gcvP2* | Glycine cleavage system protein P2 | -4.5 |
| PA2446 | *gcvH2* | Glycine cleavage system protein H2 | -3.3 |
| PA2453 | *---* | Hypothetical protein | 2. |
| PA2567 | *---* | Hypothetical protein | 2.4 |
| PA2570 | *pa1L* | PA-I galactophilic lectin | -2.1 |
| PA2575 | *---* | Hypothetical protein | -2.3 |
| PA2630 | *---* | Hypothetical protein | 4.6 |
| PA2662 | *---* | Hypothetical protein | 2.7 |
| PA2663 | *ppyR* | Psl and pyoverdine operon regulator, PpyR | 2.7 |
| PA2753 | *---* | Hypothetical protein | 4.5 |
| PA2754 | *---* | Hypothetical protein | 3.5 |
| PA2805 | *---* | Hypothetical protein | 2.5 |
| PA2851 | *efp* | Translation elongation factor P | 2 |
| PA2918 | *---* | Probable short-chain dehydrogenase | -2.3 |
| PA2939 | *---* | Probable aminopeptidase | -5.7 |
| PA2968 | *fabD* | Malonyl-CoA-[acyl-carrier-protein] transacylase | -2.1 |
| PA3006 | *psrA* | Transcriptional regulator PsrA | -2.3 |
| PA3038 | *---* | Probable porin | 2.2 |
| PA3054 | *---* | Hypothetical protein | 3.5 |
| PA3068 | *gdhB* | NAD-dependent glutamate dehydrogenase | -2.1 |
| PA3278 | *---* | Hypothetical protein | 4 |
| PA3337 | *rfaD* | ADP-L-glycero-D-mannoheptose 6-epimerase | 4.1 |
| PA3405 | *hasE* | Metalloprotease secretion protein | -3 |
| PA3406 | *hasD* | Transport protein HasD | -3.2 |
| PA3407 | *hasAp* | Heme acquisition protein HasAp | -11.2 |
| PA3408 | *hasR* | Haem uptake outer membrane receptor | -4.3 |
| PA3416 | *---* | Probable pyruvate dehydrogenase E1 component, beta chain | -2.2 |
| PA3417 | *---* | Probable pyruvate dehydrogenase E1 component, alpha subunit | -2.3 |
| PA3418 | *ldh* | Leucine dehydrogenase | -2.6 |
| PA3458 | *---* | Probable transcriptional regulator | 3.2 |
| PA3472 | *---* | Hypothetical protein | 3.6 |
| PA3478 | *rhlB* | Rhamnosyltransferase chain B | -4.5 |
| PA3479 | *rhlA* | Rhamnosyltransferase chain A | -3.3 |
| PA3520 | *---* | Hypothetical protein | -8.4 |
| PA3572 | *---* | Hypothetical protein | 3.5 |
| PA3600 | *---* | Hypothetical protein | -23.2 |
| PA3601 | *---* | Hypothetical protein | -17.5 |
| PA3613 | *---* | Hypothetical protein | 4.7 |
| PA3614 | *---* | Hypothetical protein | 2.4 |
| PA3621 | *fdxA* | Ferredoxin I | 4.1 |
| PA3661 | *---* | Hypothetical protein | 3 |
| PA3692 | *lptF* | Lipotoxin F, LptF | -2.1 |
| PA3723 | *---* | Probable FMN oxidoreductase | -2.3 |
| PA3814 | *iscS* | L-cysteine desulfurase (pyridoxal phosphate-dependent) | -2.3 |
| PA3815 | *iscR* | IscR | -2.5 |
| PA3839 | *---* | Probable sodium:sulfate symporter | 3.3 |
| PA3840 | *---* | Hypothetical protein | 2.5 |
| PA3842 | *spcS* | Specific Pseudomonas chaperone for ExoS, SpcS | 3.2 |
| PA3843 | *---* | Hypothetical protein | 2.8 |
| PA3859 | *---* | Carboxylesterase | 3.4 |
| PA3879 | *narL* | Two-component response regulator NarL | 4.7 |
| PA3880 | *---* | Hypothetical protein | 3.8 |
| PA3899 | *---* | Probable sigma-70 factor, ECF subfamily | -2.4 |
| PA3911 | *---* | Hypothetical protein | 2.3 |
| PA3912 | *---* | Hypothetical protein | 2.3 |
| PA3913 | *---* | Probable protease | 4 |
| PA3922 | *---* | Hypothetical protein | -2.9 |
| PA3952 | *---* | Hypothetical protein | 2.5 |
| PA4131 | *---* | Probable iron-sulfur protein | 2.1 |
| PA4139 | *---* | Hypothetical protein | -5.6 |
| PA4141 | *---* | Hypothetical protein | -5.2 |
| PA4155 | *---* | Hypothetical protein | -2.6 |
| PA4156 | *---* | Probable TonB-dependent receptor | -5.5 |
| PA4175 | *prpL* | Pvds-regulated endoprotease | -5.5 |
| PA4217 | *phzS* | Flavin-containing monooxygenase | -3.7 |
| PA4218 | *ampP* | AmpP | -3.1 |
| PA4219 | *ampO* | AmpO | -3 |
| PA4220 | *---* | Hypothetical protein | -4.1 |
| PA4221 | *fptA* | Fe(III)-pyochelin outer membrane receptor precursor | -4 |
| PA4225 | *pchF* | Pyochelin synthetase | -2.5 |
| PA4226 | *pchE* | Dihydroaeruginoic acid synthetase | -3 |
| PA4228 | *pchD* | Pyochelin biosynthesis protein PchD | -3.4 |
| PA4229 | *pchC* | Syochelin biosynthetic protein PchC | -3.3 |
| PA4230 | *pchB* | Salicylate biosynthesis protein PchB | -3 |
| PA4231 | *pchA* | Balicylate biosynthesis isochorismate synthase | -3 |
| PA4235 | *bfrA* | bacterioferritin | 3.3 |
| PA4294 | *---* | Hypothetical protein | -3.3 |
| PA4296 | *pprB* | Two-component response regulator, PprB | -2.2 |
| PA4299 | *tadD* | TadD | -4.2 |
| PA4300 | *tadC* | TadC | -2.8 |
| PA4301 | *tadB* | TadB | -2.3 |
| PA4302 | *tadA* | TadA ATPase | -2.7 |
| PA4303 | *tadZ* | TadZ | -2 |
| PA4304 | *rcpA* | RcpA | -2.3 |
| PA4305 | *rcpC* | RcpC | -2.2 |
| PA4306 | *flp* | Type IVb pilin, Flp | -17 |
| PA4317 | *---* | Hypothetical protein | 2.3 |
| PA4328 | *---* | Hypothetical protein | 4 |
| PA4335 | *---* | Hypothetical protein | 2.3 |
| PA4336 | *---* | Hypothetical protein | 2.2 |
| PA4348 | *---* | Hypothetical protein | 5.1 |
| PA4352 | *---* | Hypothetical protein | 4.1 |
| PA4385 | *groEL* | GroEL protein | 2.1 |
| PA4386 | *groES* | GroES protein | 2.1 |
| PA4387 | *---* | Hypothetical protein | 2.7 |
| PA4429 | *---* | Probable cytochrome c1 precursor | 2.3 |
| PA4431 | *---* | Probable iron-sulfur protein | 2.2 |
| PA4467 | *---* | Hypothetical protein | -7.1 |
| PA4468 | *sodM* | Superoxide dismutase | -6.6 |
| PA4469 | *---* | Hypothetical protein | -5.5 |
| PA4470 | *fumC1* | Fumarate hydratase | -5.9 |
| PA4494 | *roxS* | RoxS | 2.6 |
| PA4525 | *pilA* | Type 4 fimbrial precursor PilA | -49.1 |
| PA4531 | *---* | Hypothetical protein | 2.2 |
| PA4542 | *clpB* | ClpB protein | 2.9 |
| PA4570 | *---* | Hypothetical protein | -5.9 |
| PA4571 | *---* | Probable cytochrome c | 4.1 |
| PA4577 | *---* | Hypothetical protein | 3.4 |
| PA4587 | *ccpR* | Cytochrome c551 peroxidase | 8.1 |
| PA4607 | *---* | Hypothetical protein | -2.4 |
| PA4610 | *---* | Hypothetical protein | 3.6 |
| PA4615 | *---* | Probable oxidoreductase | -2.5 |
| PA4648 | *cupE1* | Pilin subunit CupE1 | -4.4 |
| PA4651 | *cupE4* | Pilin assembly chaperone CupE4 | -3.2 |
| PA4683 | *---* | Hypothetical protein | -4.2 |
| PA4702 | *---* | Hypothetical protein | -2 |
| PA4753 | *---* | Hypothetical protein | 2.3 |
| PA4761 | *dnaK* | DnaK protein | 2.2 |
| PA4762 | *grpE* | Heat shock protein GrpE | 4.1 |
| PA4825 | *mgtA* | Mg^2+^ transport ATPase. P-type 2 | 2.3 |
| PA4834 | *---* | Hypothetical protein | -6 |
| PA4843 | *---* | Probable two-component response regulator | -2 |
| PA4870 | *---* | Hypothetical protein | 2 |
| PA4874 | *---* | Hypothetical protein | -2.7 |
| PA4888 | *desB* | Acyl-CoA delta-9-desaturase, DesB | 4.2 |
| PA4889 | *---* | Probable oxidoreductase | 3.9 |
| PA4929 | *---* | Hypothetical protein | -2.1 |
| PA5023 | *---* | Hypothetical protein | -2 |
| PA5025 | *metY* | Homocysteine synthase | 3.5 |
| PA5027 | *---* | Hypothetical protein | 2.3 |
| PA5030 | *---* | Probable major facilitator superfamily (MFS) transporter | -2.7 |
| PA5049 | *rpmE* | 50S ribosomal protein L31 | 2.9 |
| PA5051 | *argS* | Arginyl-tRNA synthetase | 4.1 |
| PA5052 | *---* | Hypothetical protein | 2.6 |
| PA5053 | *hslV* | Heat shock protein HslV | 4.4 |
| PA5054 | *hslU* | Heat shock protein HslU | 3.2 |
| PA5109 | *---* | Hypothetical protein | 2.1 |
| PA5110 | *fbp* | Fructose-1.6-bisphosphatase | 2.6 |
| PA5111 | *gloA3* | Lactoylglutathione lyase | 2.3 |
| PA5174 | *---* | Probable beta-ketoacyl synthase | -3.3 |
| PA5207 | *---* | Probable phosphate transporter | 2.9 |
| PA5208 | *---* | Hypothetical protein | 4.2 |
| PA5213 | *gcvP1* | **G**lycine cleavage system protein P1 | -2 |
| PA5231 | *---* | Probable ATP-binding/permease fusion ABC transporter | 3.1 |
| PA5232 | *---* | Hypothetical protein | 3.6 |
| PA5300 | *cycB* | Cytochrome c5 | 2.2 |
| PA5415 | *glyA1 /glyA2* | Serine hydroxymethyltransferase | -3.5 |
| PA5427 | *adhA* | Alcohol dehydrogenase | 4.4 |
| PA5429 | *aspA* | Aspartate ammonia-lyase | -2 |
| PA5440 | *---* | Probable peptidase | 2.3 |
| PA5460 | *---* | Hypothetical protein | -2.3 |
| PA5470 | *---* | Probable peptide chain release factor | 7.8 |
| PA5471 | *---* | Hypothetical protein | 6.3 |
| PA5475 | *---* | Hypothetical protein | 3.1 |
| PA5479 | *gltP* | Proton-glutamate symporter | 3.1 |
| PA5495 | *thrB* | Homoserine kinase | 2.2 |
| PA5535 | *---* | Hypothetical protein | -2.8 |
