# Supplementary material for: The Extra-Cytoplasmic Function Sigma Factor SigX Modulates Biofilm and Virulence-Related Properties in Pseudomonas aeruginosa
Source: PLoS One. 2013 Nov 18;8(11):e80407. doi: 10.1371/journal.pone.0080407 (PMC3832394; doi:10.1371/journal.pone.0080407)
Supplement: Table S2 — Primer sequences of the indicated genes used for quantitative RT-PCR reactions. (DOC) [file pone.0080407.s004.doc]

**Supplementary Table S2: Primer sequences of the indicated genes used for quantitative RT-PCR reactions.**

| **PA number** | **Gene name** | **Primer name** | **Sequence (5’- 3’)** |
| --- | --- | --- | --- |
| PA0044 | *exoT* | FPA0044 | CAAGCAGATGATGCTGCAAAA |
|  |  | RPA0044 | AGCCTCTCCGCTGTCAAAGTC |
| PA0085 | *hcp1* | FPA0085 | GCAGGCGAAGCTGACCAT |
|  |  | RPA0085 | CACCGAGGACACCAGGACTT |
| PA0409 | *pilH* | FPA0409 | GGCTCGTATTTTGATTGTTGATGA |
|  |  | RPA0409 | GCCTTGAGTACCTGGTGACCAT |
| PA0527 | *dnr* | FPA0527 | TTCCGGCTGCGTGAAGAT |
|  |  | RPA0527 | GCCTCGGCGAAGGTGTT |
| PA0779 | *asrA* | FPA0779 | CCGCTTCAGTCCCAACGA |
|  |  | RPA0779 | GCGGCAAGACCTTCTCCAT |
| PA0958 | *oprD* | FPA0958 | CTACGGCTACGGCGAGGAT |
|  |  | RPA0958 | CACGTACTTGGCTTCGAGGTT |
| PA1544 | *anr* | FPA1544 | CTGACCGTGGAAGACATGGATT |
|  |  | RPA1544 | ATCACCCTGGCGGAACAG |
| PA1546 | *hemN* | FPA1546 | CGGAGGAGACCCGTACCAT |
|  |  | RPA1546 | TCTGTTTCGGCAGTCCGTAGA |
| PA1148 | *toxA* | FPA1148 | CCGGTGCGCTACAGCTACA |
|  |  | RPA1148 | TCAGTTCGTGGATGAACACCTT |
| PA1317 | *cyoA* | FPA1317 | GTCACCATCCAGGCCGTTT |
|  |  | RPA1317 | CGGGAAGGCGATCTCGTT |
| PA1706 | *pcrV* | FPA1706 | ATGCGCTGCTGAGCAATCT |
|  |  | RPA1706 | TGAGTTCCCCGCTCTGCTT |
| PA1774 | *crfX* | FPA1774 | CTGCGGGACCTGCTCAAG |
|  |  | RPA1774 | GCCGACCTGGCGATTG |
| PA1775 | *cmpX* | FPA1775 | GGGCAGATCATTGCAGGAA |
|  |  | RPA1775 | TCTCTTCAATAGTGCCTTCAACGT |
| PA1776 | *sigX* | FPA1776 | AATTGATGCGGCGTTACCA |
|  |  | RPA1776 | CCAGGTAGCGGGCACAGA |
| PA1777 | *oprF* | FPA1777 | GCGTACAGCTGGACGTGAAG |
|  |  | RPA1777 | TTCATGAAGTCAGCCAGGTTCTT |
| PA2018 | *mexX* | FPA2018 | CCATGCGTGCCCTGTTC |
|  |  | RPA2018 | ATCGCCTGCGGGTTCAC |
| PA3006 | *psrA* | FPA3006 | GGATTTCGGCGTGAACACTT |
|  |  | RPA3006 | CGAAGAACGGCACCATCAG |
| PA3405 | *hasE* | FPA3405 | TGGACGAGAAGCGTTTTTCC |
|  |  | RPA3405 | GAGACCCCGACACCCTTGT |
| PA3479 | *rhlA* | FPA3479 | GATCGAGCTGGACGACAAGTC |
|  |  | RPA3479 | GCTGATGGTTGCTGGCTTTC |
| PA3879 | *narL* | FPA3879 | CGACGGCTACCTGCTCAAG |
|  |  | RPA3879 | TGCGGGCTGAGGGTCAT |
| PA4231 | *pchA* | FPA4231 | GCCAAGGACAGGCACGAAC |
|  |  | RPA4231 | ATCGGCGTGTTCAGGTGCT |
| PA4296 | *pprB* | FPA4296 | TCGCCGAATCGCTGAACT |
|  |  | RPA4296 | TGCCGTGTACCTCGTACTTGAT |
| PA4306 | *flp* | FPA4306 | GCAAACGCCATCGAATACG |
|  |  | RPA4306 | TGGGACTCAATACGGCAATCA |
| PA4525 | *pilA* | FPA4525 | ACCCGCTGAAGACCACTGTT |
|  |  | RPA4525 | CGGTCGCAGTAGAAGCAGTAGTAC |
| 16sRNA |  | F16SRNA | AACCTGGGAACTGCATCCAA |
|  |  | R16SRNA | CTTCGCCACTGGTGTTCCTT |
